# Assessment of Plasmodium falciparum Artemisinin Resistance Independent of *kelch13* Polymorphisms and with Escalating Malaria in Bangladesh

**DOI:** 10.1128/mbio.03444-21

**Published:** 2022-01-25

**Authors:** Maisha Khair Nima, Angana Mukherjee, Saiful Arefeen Sazed, Muhammad Riadul Haque Hossainey, Ching Swe Phru, Fatema Tuj Johora, Innocent Safeukui, Anjan Saha, Afsana Alamgir Khan, Aung Swi Prue Marma, Russell E. Ware, Narla Mohandas, Barbara Calhoun, Rashidul Haque, Wasif Ali Khan, Mohammad Shafiul Alam, Kasturi Haldar

**Affiliations:** a Boler-Parseghian Center for Rare and Neglected Diseases, University of Notre Dame, Notre Dame, Indiana, USA; b Eck Institute of Global Health, University of Notre Dame, Notre Dame, Indiana, USA; c Department of Biological Sciences, University of Notre Dame, Notre Dame, Indiana, USA; d Infectious Diseases Division, International Centre for Diarrheal Diseases Research, Bangladesh (icddr,b), Dhaka, Bangladesh; e National Malaria Elimination & Aedes Transmitted Diseases Control Program, Directorate General of Health Services, Dhaka, Bangladesh; f Civil Surgeon’s Office, Bandarban, Bangladesh; g Division of Hematology, Department of Pediatrics, Cincinnati Children’s Hospital Medical Center, Cincinnati, Ohio, USA; h Global Health Center, Cincinnati Children’s Hospital Medical Center, Cincinnati, Ohio, USA; i New York Blood Center, New York, New York, USA; Rutgers−New Jersey Medical School

**Keywords:** artemisinin, Bangladesh, antimalarial agents, malaria

## Abstract

Emerging resistance to artemisinin drugs threatens the elimination of malaria. Resistance is widespread in South East Asia (SEA) and Myanmar. Neighboring Bangladesh, where 90% of infections occur in the Chittagong Hill Tracts (CHTs), lacks recent assessment. We undertook a prospective study in the sole district-level hospital in Bandarban, a CHT district with low population densities but 60% of reported malaria cases. Thirty patients presented with malaria in 2018. An increase to 68 patients in 2019 correlated with the district-level rise in malaria, rainfall, humidity, and temperature. Twenty-four patients (7 in 2018 and 17 in 2019) with uncomplicated Plasmodium falciparum monoinfection were assessed for clearing parasites after starting artemisinin combination therapy (ACT). The median (range) time to clear half of the initial parasites was 5.6 (1.5 to 9.6) h, with 20% of patients showing a median of 8 h. There was no correlation between parasite clearance and initial parasitemia, blood cell counts, or mutations of P. falciparum gene *Pfkelch13* (the molecular marker of artemisinin resistance [AR]). The *in vitro* ring-stage survival assay (RSA) revealed one (of four) culture-adapted strains with a quantifiable resistance of 2.01% ± 0.1% (mean ± standard error of the mean [SEM]). Regression analyses of *in vivo* and *in vitro* measurements of the four CHT strains and WHO-validated K13 resistance mutations yielded good correlation (*R*^2^ = 0.7; ρ = 0.9, *P* < 0.005), strengthening evaluation of emerging AR with small sample sizes, a challenge in many low/moderate-prevalence sites. There is an urgent need to deploy multiple, complementary approaches to understand the evolutionary dynamics of the emergence of P. falciparum resistant to artemisinin derivatives in countries where malaria is endemic.

## INTRODUCTION

**FIG 1:**
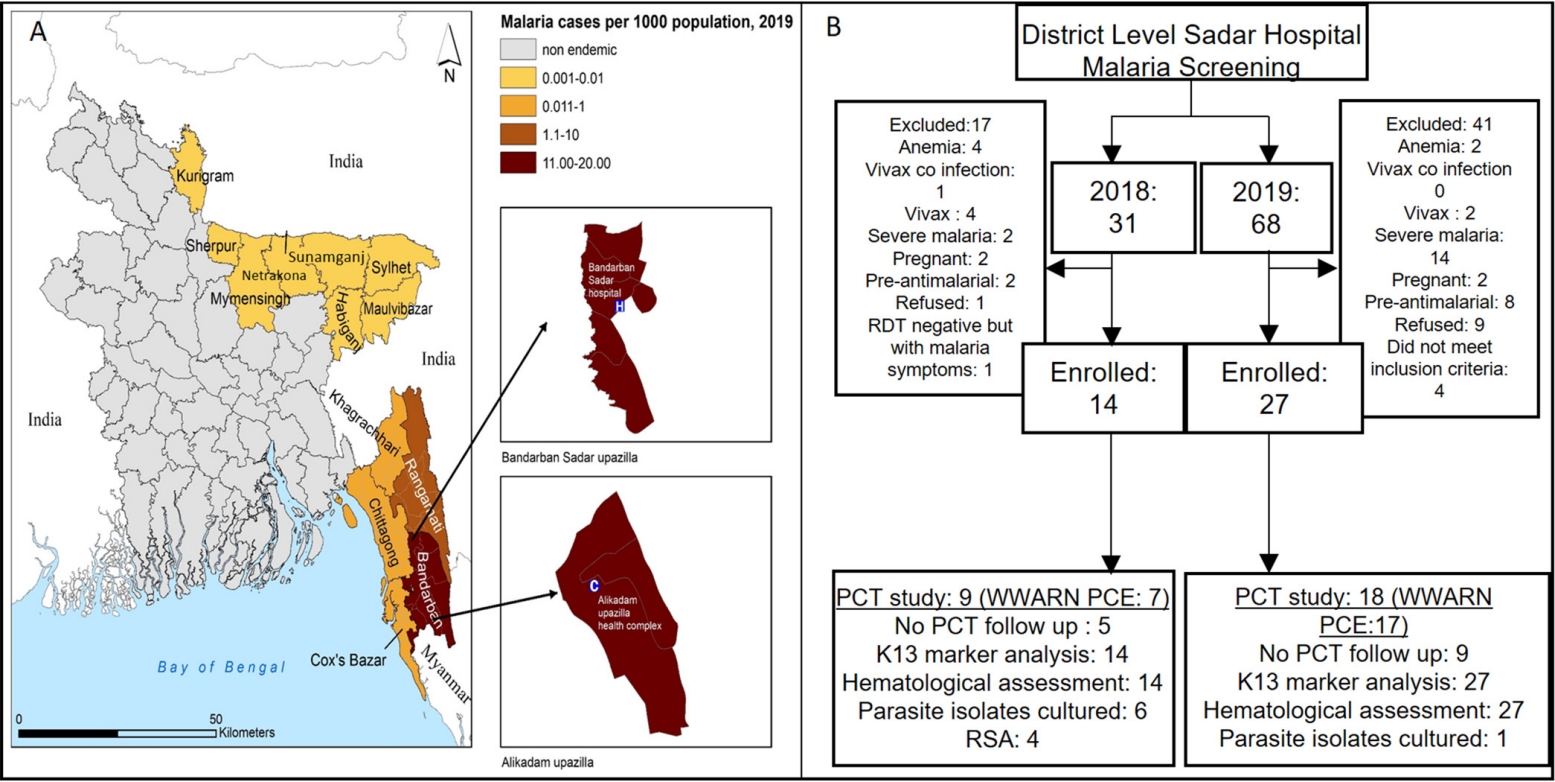
Study sites and study flow chart. (A) Map of Bangladesh highlighting the regions where malaria is endemic, with two submaps showing the Bandarban Sadar and Alikadam Upazila study sites. Data were provided by the National Malaria Elimination Program Bangladesh (NMEP). (B) Bandarban study flow chart. In 2018, 7 of 9 PCT profiles could be evaluated by the WWARN PCE tool. In 2019, 17 of 18 PCT profiles could be evaluated by the WWARN PCE tool.

Malaria has taken a staggering historical toll on human health. However, in the 21st century, many areas of the world are striving for elimination and eradication. From 2010 to 2015, malaria cases declined globally (1). Fast-acting artemisinin combination therapies (ACTs) reduced estimated parasitemias and death due to Plasmodium falciparum, the most virulent of human malaria parasites responsible for the major burden of disease. Yet, over 400,000 deaths and 228 million worldwide cases of malaria occurred in 2018 (1) and global malaria levels have failed to decrease since 2015 (2). Malaria elimination in Bangladesh (where 17.5 million people live at risk for infection) made strong progress from 2010 to 2018, with malaria cases reduced by 81% (https://nmcp.gov.bd). Over 90% of malaria cases occur in the Chittagong Hill Tracts (CHTs) in three districts, Khagrachhari, Rangamati, and Bandarban, which border Myanmar (see Fig. 1A).

Drugs are the mainstay for eliminating blood stages of parasites that are responsible for all the signs and symptoms of malaria. Resistance to drugs therefore threatens malaria control. Resistance to chloroquine arose in South East Asia (SEA), spreading from South Asia with devastating effects for malaria morbidity/mortality in Africa 30 years ago (3). The more recent spread of resistance to frontline artemisinins throughout SEA and Myanmar has led to concern that there may be spread to Bangladesh, which in turn may lie in the path of global dissemination of resistance (4–6). Artemisinin resistance (AR) may also arise independently (7–10), suggesting multiple ways that Bangladesh parasites may become refractory to these frontline drugs for which we still have no replacements.

Resistance to artemisinins was first reported in clinical studies as a delay in clearance of early blood-stage (or “ring-stage”) infection from patient circulation, in response to drug treatment (11, 12). Subsequent studies established P. falciparum *kelch13* (K13) as a molecular and major causal marker of AR in P. falciparum malaria (13, 14). Reports from SEA have established a strong association between polymorphisms in the β-propeller domain of K13 and delayed clearance of parasites in circulation (15). But, in areas of high transmission, immunity might mask delayed clearance (7, 16). Polymorphisms in K13 and additional genes associated with resistance have been characterized by the laboratory-based ring-stage survival assay (RSA) (17), an *in vitro* correlate of *in vivo* resistance. This laboratory assay is important for proving that mutations in candidate genes when engineered into sensitive parasites in local genetic or laboratory strains are causative for resistance (18, 19) as well as strains where resistance is mediated by unknown determinants (20).

Detection of mutations in K13 (as well as other drug resistance markers) by application of PCR-based technologies to infected blood from patients is well established and widely utilized in Bangladesh (21, 22) However, measurements of *in vivo* parasite clearance in the CHTs have not been done. A major challenge is that CHTs are remote and rural (Fig. 1A). Here, we present the first report of successful adaptation and establishment of a clinical assay of P. falciparum clearance at a district-level hospital in the CHTs. We also established *in vitro* cultures of strains isolated from the CHTs, report the first execution of RSA to evaluate *in vitro* AR in Bangladesh strains, and show that multiple, advanced complementary approaches are needed to assess AR in the context of the sharp escalation of malaria associated with climatic factors.

## RESULTS

### Overall clinical presentation profiles.

*In vivo* parasite clearance studies in Bandarban Sadar Hospital were undertaken over 2 years, from 2018 to 2019 (Fig. 1). In 2018, of 31 patients who presented with malaria symptoms, 17 were excluded because they were either infected or coinfected with Plasmodium vivax, showed severe malaria, were anemic or pregnant, had recent prior exposure to antimalarials, refused to consent, or were rapid diagnostic test (RDT) negative (as found for one patient in 2018) despite having malaria symptoms (also summarized in Fig. 1B). Of the 14 patients with P. falciparum monoinfections, 9 were enrolled in parasite clearance time (PCT) studies. The remaining 5 showed initial parasitemias below 1,000 parasites/μL, the threshold level for inclusion in PCT measurements. However, all 14 were subjected to K13 marker analyses and hematological assessments. Four were evaluated in the RSA. In 2019, all 68 patients who presented with malaria symptoms were RDT positive, and 27 met inclusion criteria of P. falciparum monoinfection without additional factors. Eighteen who showed an initial parasitemia of ≥1,000 parasites/μL were enrolled in PCT studies (but all 27 were included in K13 and hematological analyses).

**FIG 2:**
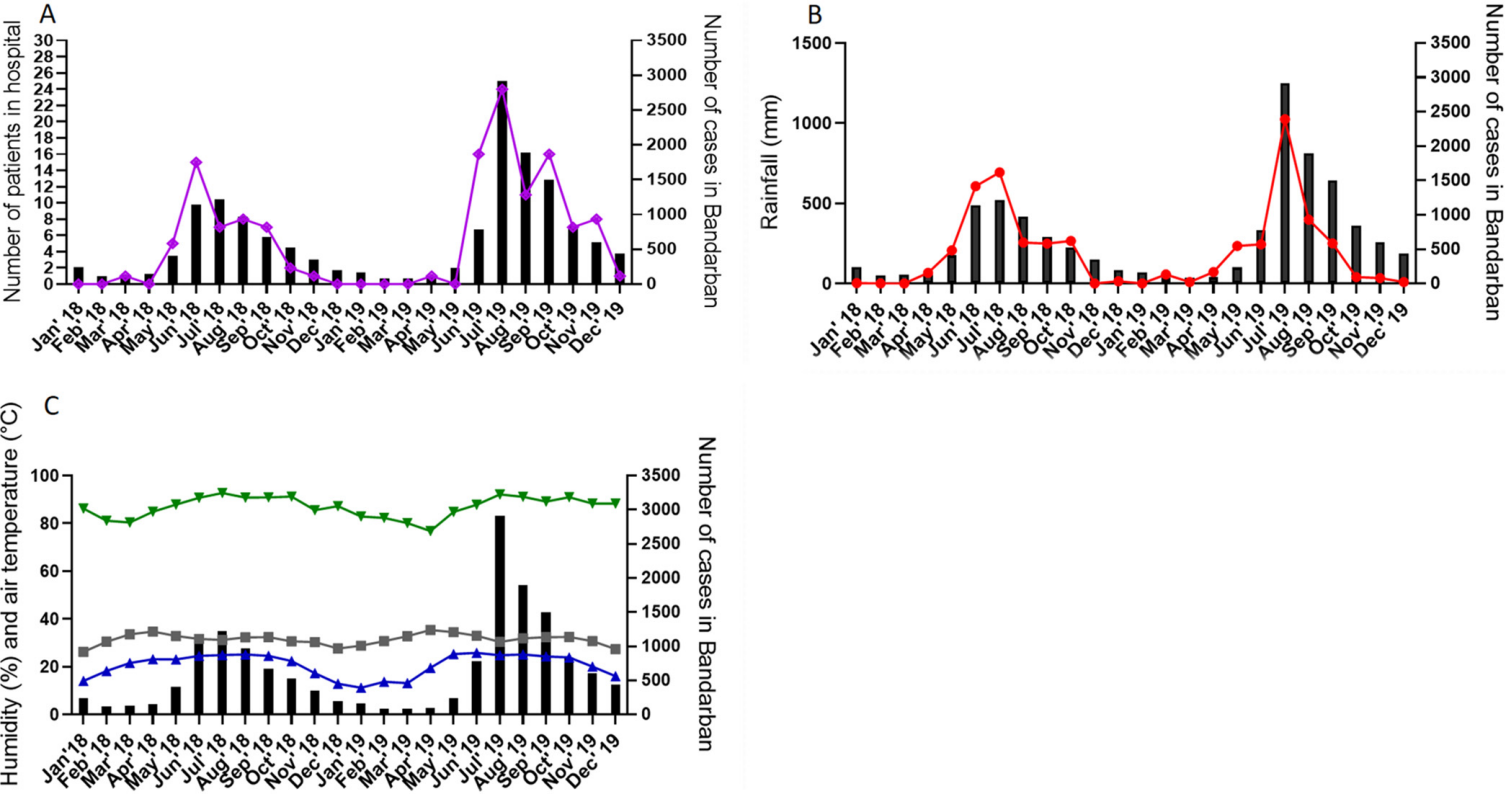
Comparative analyses of malaria patients admitted to Bandarban Sadar Hospital, total malaria incidence, and associations with climate factors in Bandarban District, 2018 to 2019. (A) Total monthly malaria cases in Bandarban Sadar Hospital (purple line) and Bandarban District (black bars) (Spearman coefficient, ρ = 0.8804, *P* < 0.0001). (B) Total monthly malaria cases (black bars) and rainfall (red line) in district (Spearman coefficient, ρ = 0.73, *P* < 0.0001). (C) Total district monthly malaria cases and maximum average temperature distribution (gray squares) (Spearman coefficient, ρ = −0.12, *P* = 0.58), minimum average temperature (blue triangles) (Spearman coefficient, ρ = 0.72, *P* = 0.0001), and average humidity (green triangles) (Spearman coefficient, ρ = 0.91, *P* < 0.0001). Annual parasite index (API) in Bandarban Sadar was 0.83 (population at risk, *n* = 152,915) in 2018 and 1.60 (population at risk, *n* = 156,738) in 2019.

Together, the data in Fig. 1 suggested that although P. falciparum infections were dominant, only about a third could be utilized for PCT studies due to other complications of presentations or insufficient threshold parasitemia. One unexpected observation was that the number of malaria patients presenting at the clinic increased by ∼2-fold from 2018 to 2019 (Fig. 1B). We took this in the context that many levels of vector-borne diseases like malaria can be affected by climate and that Bangladesh is known to be vulnerable to climate change. As shown in Fig. 2A, monthly presentation of malaria patients in the clinic closely corresponded with the monthly parasite incidence levels in Bandarban district (Spearman correlation coefficient, ρ = 0.8804, *P* < 0.0001), which in turn showed significant correlation to rainfall (Spearman coefficient, ρ = 0.73, *P* < 0.0001) (Fig. 2B), humidity (Spearman coefficient, ρ = 0.91, *P* < 0.0001) (Fig. 2C), and minimum temperature (Spearman coefficient, ρ = 0.72, *P* = 0.0001) (Fig. 2C). This strongly suggested that the 2-fold increase seen in presenting patients in 2019 was due to higher levels of malaria in the district rather than other determinants affecting access and care.

Participants enrolled in PCT studies were young adults, with 67% males (Table 1). At the time of presentation, all but one had fever (≥37.5°C), and none were significantly anemic (hemoglobin [Hb], <9 g/dL for females and 10 g/dL for males) (Table 1; see Table S1 and Data Set S1 in the supplemental material). All participants tolerated and complied with study procedures. No serious adverse events were reported. Mild to moderate adverse events of headache, body aches, nausea, vomiting, fever, and/or chill were observed and recorded for each participant. They were related to acute malaria illness and resolved within 72 h or 3 days. Participants did not receive acetaminophen or other antipyretics after the first dose of the ACT artemether-lumefantrine (Komefan 140).

**TABLE 1:** Presenting clinical characteristics and parameters of participants enrolled in PCT studies in Bandarban District Hospital, Sadar, 2018 to 2019^*a*^

Parameter^*b*^	Median value (IQR) in indicated yr(s)		
	2018	2019	Combined (2018 and 2019)
No. of patients∗	9	18	27
Age (yr)	29 (15–45)	17.50 (13.75–42.75)	24 (14–45)
Gender (%)	Male (66.67)	Male (66.67)	Male (66.67)
Temp at enrollment (°C)	38 (36.6–39.4)	37.7 (37.2–38.3)	38.8 (37.7–39.4)
Initial parasite density (per μL)	22,280 (8,760–152,500)	18,900 (9,790–64,829)	20,020 (9,840–116,875)
**Geometric mean (95% CI)**∗	**25,781 (7,458–89,122)**	**23,000 (11,322–46,724)**	**23,892 (13,408–42,572)**
Parasitemia (%)	0.52 (0.2–2.8)	0.34 (0.18–1.5)	0.34 (0.17–2.2)
Hemoglobin (%) (g/dL)			
Male	13.7 (11.2–15.3)	11.6 (10.6–13.4)	12.2 (10.9–14)
Female	10.7 (9.4–11.9)	10.9 (9.5–13.7)	10.7 (9.5–12.7)

a See also Table S1 in the supplemental material. Parasite density geometric means are indicated in bold.

b ∗, values do not represent median (IQR).

10.1128/mbio.03444-21.1 DATA SET S1 All patient parameters associated with clinical studies. Download Data Set S1, PDF file, 1.9 MB. Copyright © 2022 Nima et al. 2022 Nima et al. https://creativecommons.org/licenses/by/4.0/ This content is distributed under the terms of the Creative Commons Attribution 4.0 International license.

10.1128/mbio.03444-21.2 TABLE S1 Participant baseline parameters at time of enrollment in the study in Bandarban District Hospital, Sadar, 2018 to 2019. Age, gender, febrile status, parasite density (per μL), parasitemia (%), and hemoglobin (g/dL) data are shown. Download Table S1, PDF file, 0.3 MB. Copyright © 2022 Nima et al. 2022 Nima et al. https://creativecommons.org/licenses/by/4.0/ This content is distributed under the terms of the Creative Commons Attribution 4.0 International license.

### Clinical outcomes and parasite clearance.

**FIG 3:**
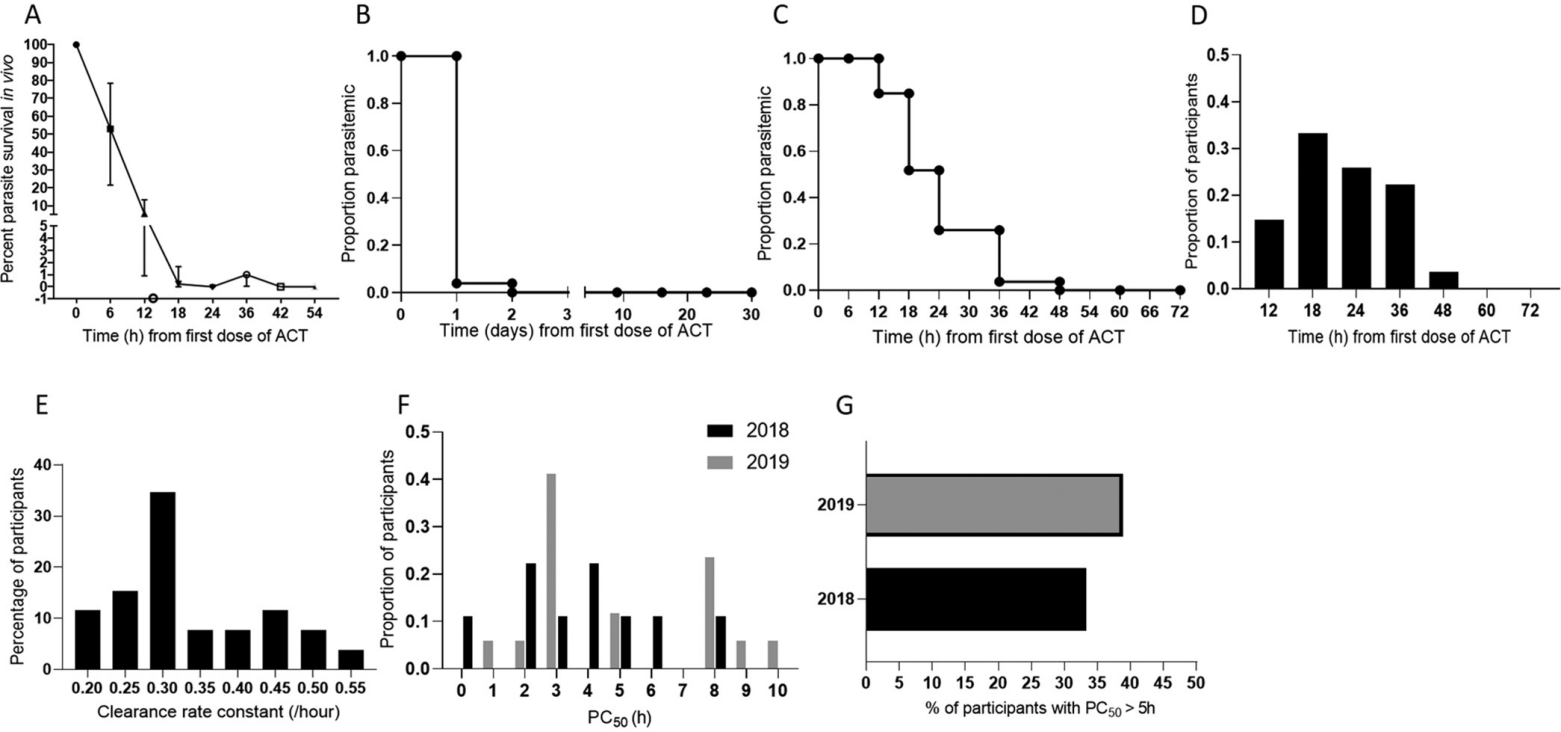
Parasite clearance data, cumulative proportion of parasitemic patients, clearance over time, and rate and time to clear half the initial parasites (PC_50_). (A) Parasite clearance curve showing linear regression of percent parasite density *in vivo* over time (hours), with median and interquartile range (IQR) shown. (B) Cumulative proportion of parasitemic participants and their clearance over a 30-day period (follow-up on days 9, 16, 23, and 30). (C) Cumulative proportion of parasitemic participants and their clearance over 72 h after administration of first dose of ACT. (D) Proportion distribution of participants with time (hours) of first negative microscopy slide after administration of first dose of ACT. (E) Percentage of patients with indicated clearance rate constant (per hour). (F) Proportion of participants with associated PC_50_ values, samples collected in 2018 (black bars) and 2019 (gray bars). (G) Percentage of participants with delayed 50% clearance (PC_50_) in 2018 and 2019. (See also Fig. S2 in the supplemental material.)

**FIG 4:**
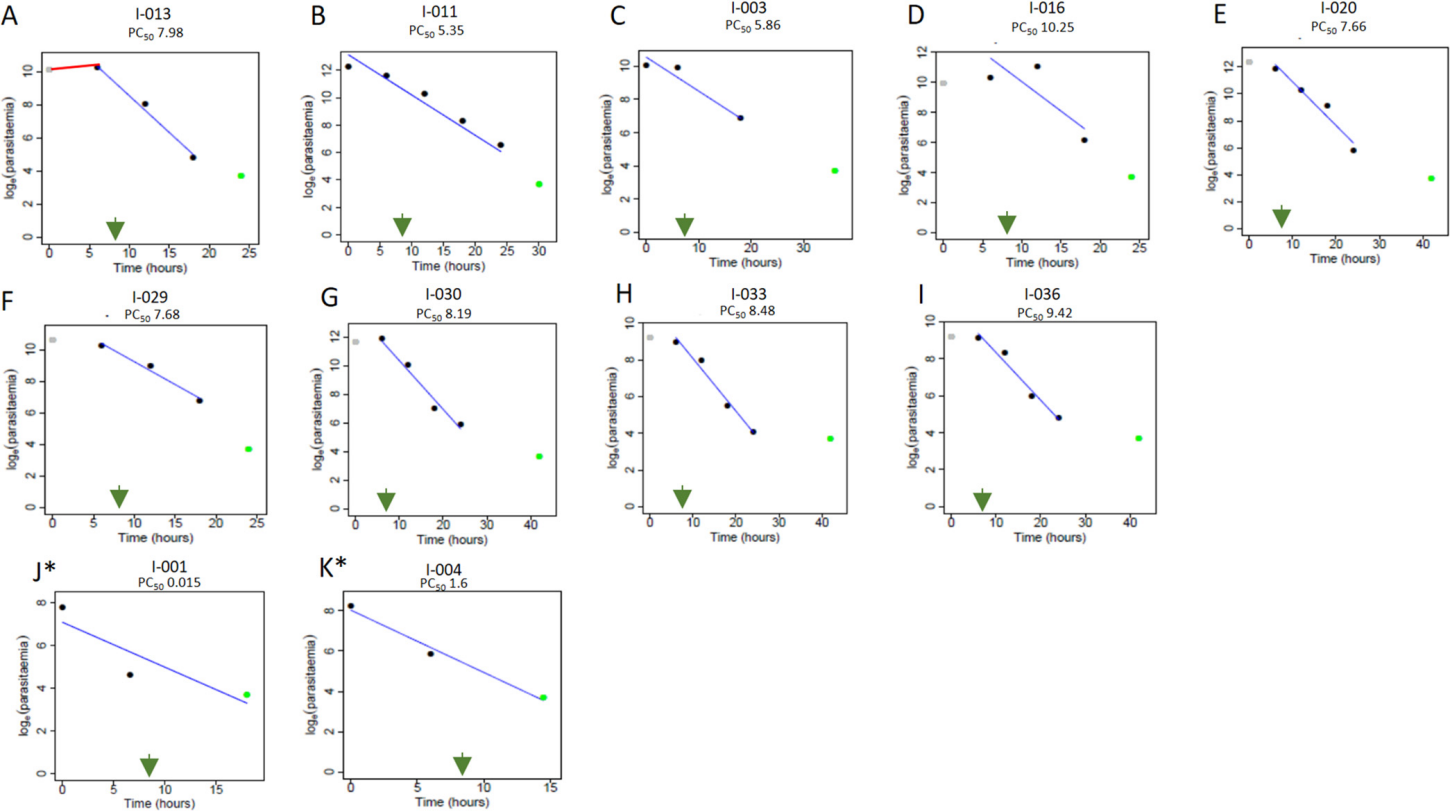
Data plots of *in vivo* parasite clearance estimation for individual participants after administration of ACT. Blood draws were at 0, 6, 12, 18, 24, and 36 h (black circles). At *t* = 0, smears were made prior to drug administration. (A to I) WWARN plots of individual 50% parasite clearance (PC_50_) values exceeding 5 h. Green circle, zero parasitemia set at log 4 to fit Tobit regression model (WWARN PCE); gray circle, lag phase measurement. For panel A, the red line shows the lag phase, annotated. In all panels, the green arrow shows the 8-h time point of the second ACT dose administration. Panels J* and K* indicate 2 (of 15) clearance profiles where there was no observed delay.

Enrollment parasite densities were moderate in each year (Table 1). Over 2 years, the geometric mean was 20,020/μL, with a 95% confidence interval of 9,840 to 116,875/μL. The median time to fever clearance was 24 h, with a range of 24 to 48 h (not shown). The aggregate of all data points was included in measurement of parasite clearance times independent of initial parasitemia (Fig. 3A; Table 2). For the 24 patients studied, the median (range) time to clear half of the initial parasites was 5.6 (1.5 to 9.6) h. The median (range) times to clear half of the parasites enrolled in 2018 and 2019 were 3.1 (1.5 to 4.8) h and 7.0 (2.9 to 9.6) h, respectively (Table 2; Fig. S1A and S1B). The WorldWide Antimalarial Resistance Network parasite clearance estimator (WWARN PCE) (23) profiles of isolates with times to clear half of the initial parasites of >5 h are provided in Fig. 4A to I. Lag phase in parasite clearance was detected in 7 (Fig. 4A and D to I) of 9 clearance curves shown. An increase in parasitemia in the lag phase (Fig. 4A, D, and G) may signify bursting of “schizont” stage parasites to release new “merozoites,” which in turn give rise to ring stages monitored in the clearance assay (24). Two clearance curves (Fig. 4B and C) were assessed not to have a lag phase by the WWARN PCE, although the rate of parasite decline in the first 6 h appeared lower than in subsequent intervals. Since the standard of care was a second dose of artemether-lumefantrine at 8 h (Fig. 4, green arrow), at the outset we did not prioritize analyses based on the half-life of the slope of parasite clearance (PC_1/2_) between 6 and 24 h. For the majority of patients (15 of 24), the time to clear half the parasites was substantially less than 5 h; two examples of such clearance curves are shown in Fig. 4J to K. The parasite clearance half-life based on the initial value and the times required to reach 50%, 90%, and 95% clearance (PC_50_, PC_90_, and PC_95_, respectively) are summarized in Table 2. All parasite infections were cleared by 72 h or 3 days after start of treatment (Fig. 3B and C).

**TABLE 2:** Parasite clearance data for study participants in 2018 and 2019

Parasite clearance parameter	Median value (IQR) (range) for indicated yr(s)		
	2018	2019	2018 and 2019
Parasite clearance time (h)^*a*^	24 (15–24) (12–36)	24 (18–36) (12–48)	24 (18–36) (12–48)
Parasite clearance half-life (h)^*b*^	3.1 (1.5–3.1) (1.5–4.8)	6.9 (3.2–7.8) (2.8–9.6)	5.6 (2.8–7.3) (1.5–9.6)
PC_50_ (h)	3.5 (1.8–5.6) (0.015–7.9)	3.9 (3.2–8.2) (1.2–8.2)	3.5 (2.8–7.6) (0.015–7.6)
PC_90_ (h)	8.42 (6.4–11.2) (5.3–13.7)	9.45 (8–13.4) (5.6–19.2)	9.04 (7.6–12.9) (5.3–19.2)
PC_95_ (h)	10.9 (8.2–13.2) (6.7–17.2)	12.44 (10.16–15.85) (6.8–21.8)	11.83 (9.6–14.96) (6.7–21.8)
PC_99_ (h)	16.8 (12.5–18.6) (10–25)	19.2 (14.6–21.4) (9.7–27.9)	17.4 (14.3–19.7) (9.6–27.9)

a Time of first observed negative microscopy slide after first dose of ACT (hours).

b Estimated time (hours) for 50% reduction in parasite density, obtained from percent parasite survival. The parasite clearance values PC_50,_ PC_90,_ PC_95_, and PC_99_ (hours) indicate time to reach 50, 90, 95, and 99% reduction in initial parasite density, respectively.

10.1128/mbio.03444-21.5 FIG S1 Parasite clearance data, cumulative proportion of parasitemic patients and their clearance over time, clearance rate, and associated 50% clearance values in patients enrolled in 2018 and 2019. The parasite clearance curve shows linear regression of percent parasite density *in vivo* over time (h) in 2018 (A) and 2019 (B). The cumulative proportion of parasitemic participants and their clearance over a 30-day period (follow-up on days 9, 16, 23, and 30) are shown for 2018 (C) and 2019 (D). The cumulative proportion of parasitemic participants and their clearance over 72 h after administration of the first dose of ACT are shown for 2018 (E) and 2019 (F). The proportion distribution of participants with time (h) of first negative microscopy slide after administration of first dose of ACT is shown for samples collected in 2018 (G) and 2019 (H). Download FIG S1, TIF file, 0.07 MB. Copyright © 2022 Nima et al. 2022 Nima et al. https://creativecommons.org/licenses/by/4.0/ This content is distributed under the terms of the Creative Commons Attribution 4.0 International license.

10.1128/mbio.03444-21.6 FIG S2 Associations between initial parasite density and hematological parameters of study participants from Bandarban District Hospital, Sadar. Linear regression and correlation analyses between initial parasite density (parasites/μL) and platelets (Spearman coefficient, ρ = −0.35, *P* = 0.02, *R*^2^ = 0.04) (A), mean corpuscular volume (MCV) (Spearman coefficient, ρ = 0.2, *P* = 0.2, *R*^2^ = 0.036) (B), mean corpuscular hemoglobin (MCH) (Spearman coefficient, ρ = 0.3, *P* = 0.1, *R*^2^ = 0.04) (C), mean corpuscular hemoglobin concentration (MCHC) (Spearman coefficient, ρ = 0.3, *P* = 0.04, *R*^2^ = 0.03) (D), white blood cell–total count (WBC-TC) (Spearman coefficient, ρ = −0.2, *P* = 0.17, *R*^2^ = no fit) (E), lymphocytes (Spearman coefficient, ρ = −0.2, *P* = 0.2, *R*^2^ = 0.11) (F), neutrophils (Spearman coefficient, ρ = 0.17, *P* = 0.3, *R*^2^ = 0.1) (G), mixed cell count (MXD), including monocytes, basophils, and eosinophils (Spearman coefficient, ρ = −0.03, *P* = 0.8, *R*^2^ = 0.008) (H). Download FIG S2, TIF file, 0.09 MB. Copyright © 2022 Nima et al. 2022 Nima et al. https://creativecommons.org/licenses/by/4.0/ This content is distributed under the terms of the Creative Commons Attribution 4.0 International license.

Since the numbers of participants were different in 2018 and 2019, we examined the proportion of participants as a function of the time in hours to a “slide-negative” state to confirm that clearance was achieved by 60 h (Fig. 3C; Fig. S1C and D). While most patients in 2018 and 2019 reported complete parasite clearance within 48 h in 2018 and 2019, one patient reported 54 h to clear all parasites, the longest time recorded for complete parasite clearance in this study (Fig. S1E to H). Histograms of the number of participants and their clearance rate constant/hour (Fig. 3D and E) suggested that the highest number of participants (34%) had a clearance rate constant of 0.30/h, followed by 11% of participants with a clearance constant rate of 0.45/h, the second highest percentage of participants. Analyses of the proportion of parasites as a function of 50% parasite clearance (PC_50_) also supported a bimodal distribution (Fig. 3F and G). This included the expected fast-clearing parasites that reach a median 50% density by 3 (2.0 to 4.0) h, but in addition, approximately 20% of parasites showed a slower median clearance of half the initial parasites at 8 (6 to 10) h. This was more marked in 2019 than in 2018. The proportion of participants per year with a PC_50_ of >5 h increased from ∼34% to 40% from 2018 to 2019, with 38% of patients having a median 50% parasite clearance of 8.4 (7.6 to 10.2) h in 2019. Together, these data suggest that an *in vivo* delay in reducing the initial P. falciparum parasitemia by 50% was reliably detected in two consecutive years, 2018 and 2019. Moreover, although the number of P. falciparum infections increased ∼2 fold, the fraction showing slower clearance to half of the initial parasites in 2019 was comparable to that in 2018. Nonetheless, all infections were cured, indicating that there was no failure of the ACT artemether-lumefantrine.

Since immunity may mask AR, we subsequently undertook regression analyses to understand parasite and host factors that may influence parasite clearance. As shown in Fig. S3, there was no major association between PC_50_ (Table 2) and potential modifiers, including age, gender, and initial parasite density in either 2018 or 2019. Additionally, regression analyses failed to yield high-confidence associations between PC_50_ and initial mean corpuscular volume (MCV), mean corpuscular hemoglobin concentration (MCHC), mean corpuscular hemoglobin (MCH), white blood cells–total count (WBC-TC), mixed cell count (MXD) (i.e., monocytes, basophils, eosinophils), and platelets (Fig. S4). Regression and correlation analyses and median values and range for all parameters are shown in Fig. S3 and Table 3, respectively. Regression and correlation analyses between initial parasite density and hematological parameters also failed to show high-confidence associations (Fig. S2). Together, the data in Fig. S2 to S4 and Table 3 suggest that initial parasitemia and blood count parameters did not make substantial contributions to *in vivo* clearance phenotypes. Patient hematological readouts are provided in Table S2 and Data Set S1.

**TABLE 3:** Hematological and immune cell characteristics presented by participants enrolled in PCT studies in Bandarban District Hospital, Sadar, 2018 to 2019^*a*^

Parameter	Median value (IQR) in indicated yr(s)		
	2018	2019	2018 and 2019
No. of participants	9	18	27
Reticulocytes (%)	0.9 (0.7–1.1)	0.9 (0.6–1.2)	0.9 (0.7–1.2)
Platelets × 10^3^ (per μL)	94 (40.5–144)	74 (51.7–120.8)	81 (48–141)
MCV (fL)	68.8 (65.2–81.7)	66 (60.6–73.3)	68.2 (61.4–76.5)
MCH (pg)	24.9 (22–28)	21.9 (19.4–25)	22. (20.10–26.2)
MCHC (g/dL)	34 (33.1–34.5)	33.25 (32.4–33.8)	33.40 (32.6–34.1)
WBC-TC × 10^3^ (per μL)	5.4 (4.7–6.7)	6.4 (4.4–7.9)	5.9 (4.5–7.3)
Lymphocytes (%)	23.3 (12.1–30.4)	20.3 (12.2–37.3)	20.9 (13–32)
Neutrophils (%)	66.5 (52.7–79.8)	68.6 (54.6–77.7)	67.2 (56.2–77.9)
MXD (%)	5.3 (4.9–19.3)	6.2 (4.5–9.3)	6.1 (5–11.1)

a See also Table S2 in the supplemental material.

10.1128/mbio.03444-21.3 TABLE S2 Hematological and blood count parameters of participants enrolled in the study in Bandarban District Hospital, Sadar, 2018 to 2019. Reticulocytes (%), platelets (per μL), mean corpuscular volume (fL), mean corpuscular hemoglobin (pg), mean corpuscular hemoglobin concentration (g/dL), and immune cell characteristics (whole blood cell–total count per μL), lymphocytes (%), neutrophils (%), and monocytes-basophils-eosinophils (%) are shown. Download Table S2, PDF file, 0.2 MB. Copyright © 2022 Nima et al. 2022 Nima et al. https://creativecommons.org/licenses/by/4.0/ This content is distributed under the terms of the Creative Commons Attribution 4.0 International license.

10.1128/mbio.03444-21.7 FIG S3 Associations between 50% parasite clearance (PC_50_) and baseline parameters. (A) Age of study participants (Spearman coefficient, ρ = −0.026, *P* = 0.89, *R*^2^ = 0.00096); (B) Mann-Whitney test of PC_50_ between male and female participants (*P*= 0.17); (C) initial parasite density, samples collected in 2018 (Spearman coefficient, ρ = 0.63, *P* = 0.076, *R*^2^ = 0.04); (D) initial parasite density, samples collected in 2019 (Spearman coefficient, ρ = 0.27, *P* = 0.28, *R*^2^ = 0.017); (E) initial parasite density, samples collected in 2018 and 2019 (Spearman coefficient, ρ = 0.37, *P* = 0.054, *R*^2^ = 0.016). Download FIG S3, TIF file, 0.04 MB. Copyright © 2022 Nima et al. 2022 Nima et al. https://creativecommons.org/licenses/by/4.0/ This content is distributed under the terms of the Creative Commons Attribution 4.0 International license.

10.1128/mbio.03444-21.8 FIG S4 Associations between 50% parasite clearance (PC_50_) and hematological parameters. (A) Hemoglobin % (g/dL) (Spearman coefficient, ρ = 0.4, *P* = 0.04, *R*^2^ = 0.08); (B) platelets (μl) (Spearman coefficient, ρ = −0.13, *P* = 0.52, *R*^2^ = 0.02); (C) mean corpuscular volume (MCV) (fL) (Spearman coefficient, ρ = 0.03, *P* = 0.8, *R*^2^ = 0.005); (D) mean corpuscular hemoglobin (MCH) (pg) (Spearman coefficient, ρ = −0.015, *P* = 0.08, *R*^2^= 0.03); (E) mean corpuscular hemoglobin concentration (MCHC) (g/dL) (Spearman coefficient, ρ = −0.19, *P* = 0.3, *R*^2^ = 0.05); (F) white blood cells–total count (WBC-TC) (μl) (Spearman coefficient, ρ = 0.37, *P* = 0.05, *R*^2^ = 0.2); (G) lymphocytes (%) (Spearman coefficient, ρ = 0.38, *P* = 0.04, *R*^2^ = 0.08); (H) neutrophils (%) (Spearman coefficient, ρ = 0.48, *P* = 0.01, *R*^2^ = 0.15); (I) mixed cell count (MXD), including monocytes, basophils, and eosinophils (%) (Spearman coefficient, ρ = −0.32, *P* = 0.4, *R*^2^ = 0.08). Download FIG S4, TIF file, 0.06 MB. Copyright © 2022 Nima et al. 2022 Nima et al. https://creativecommons.org/licenses/by/4.0/ This content is distributed under the terms of the Creative Commons Attribution 4.0 International license.

### K13 polymorphisms.

In 2018, low numbers of patients were enrolled in Bandarban Sadar due to low levels of malaria in the area. Because of this, in 2019, we added collection of parasites in Alikadam Upazila Health Complex, where the annual parasite index (API) was more than 10-fold higher than that in Bandarban Sadar.

As summarized in Fig. S5, 86 patient samples were collected with acute P. falciparum monoinfection in the absence of severe manifestation who were enrolled at Alikadam. Participants ranged from pediatric individuals to young adults. Sixty-three percent and 75% were males in 2019 and 2020, respectively. Parasite densities were moderate (2018 geometric mean, 22,460/μL; 95% confidence interval [CI], 4,860 to 35,850/μL; 2019 geometric mean, 45,000/μL; 95% confidence interval, 4,480 to 145,000/μL), and patients were not anemic (Hb < 9 g/dL). Since K13 polymorphisms associated with AR are invariably associated with the β-propeller domain, we restricted analyses to this region. As shown in Fig. S5C, there were no polymorphisms identified in Bandarban Sadar. One polymorphism, K13A578S, was detected in Alikadam. Prior studies have reported K13A578S (which fails to confer a resistant phenotype) in the CHTs (21). Together, these findings suggest that *in vivo* delay in clearance of 50% of the initial parasitemia cannot be explained by resistance mutations in the β-propeller domain of K13.

10.1128/mbio.03444-21.9 FIG S5 Study flow chart of Alikadam, baseline characteristics, and K13 polymorphism at all study sites. (A) Alikadam study flow chart. (B) Baseline characteristics, parasite clearance, and incidence parameters in participants enrolled in Alikadam Upazila Health Complex (UHC) in 2019 and 2020. (C) K13 polymorphisms in Bandarban Sadar and Alikadam Upazila. The sequence of the Bangladesh strain I-003 K13 gene (compared to that of *Pf3D7*) is provided in Data Set S1 in the supplemental material. Download FIG S5, TIF file, 0.09 MB. Copyright © 2022 Nima et al. 2022 Nima et al. https://creativecommons.org/licenses/by/4.0/ This content is distributed under the terms of the Creative Commons Attribution 4.0 International license.

### Culture adaptation and RSA.

Studies in SEA established a strong association between polymorphisms in the β-propeller domain of K13 and delayed clearance of parasites in circulation. However, polymorphisms in additional genes have also been associated with and shown to be causal for resistance, as determined in the laboratory-based RSA (17–19). As summarized in Fig. S6, we culture adapted 6 of 9 isolates obtained in 2018 and 1 of 18 in 2019. The range of initial parasitemia varied from 0.06% to 3.9%. Nonetheless, 2% parasitemia was achieved within 9 to 12 days.

10.1128/mbio.03444-21.10 FIG S6 Culture adaptation. (A) Flow chart of laboratory culture adaptation attempts in 2018 and 2019. (B) Isolates and their initial parasitemias and days taken to reach at least 2% parasitemia. Patient isolates I-001, I-003, I-004, and I-011 were forwarded to RSA. I-002 parameters could not be analyzed by the WWARN PCE. For outpatient O-012, there were no WWARN PCE data. Isolate I-017 could not be expanded in *in vitro* culture for evaluation by RSA. Download FIG S6, TIF file, 0.04 MB. Copyright © 2022 Nima et al. 2022 Nima et al. https://creativecommons.org/licenses/by/4.0/ This content is distributed under the terms of the Creative Commons Attribution 4.0 International license.

**FIG 5:**
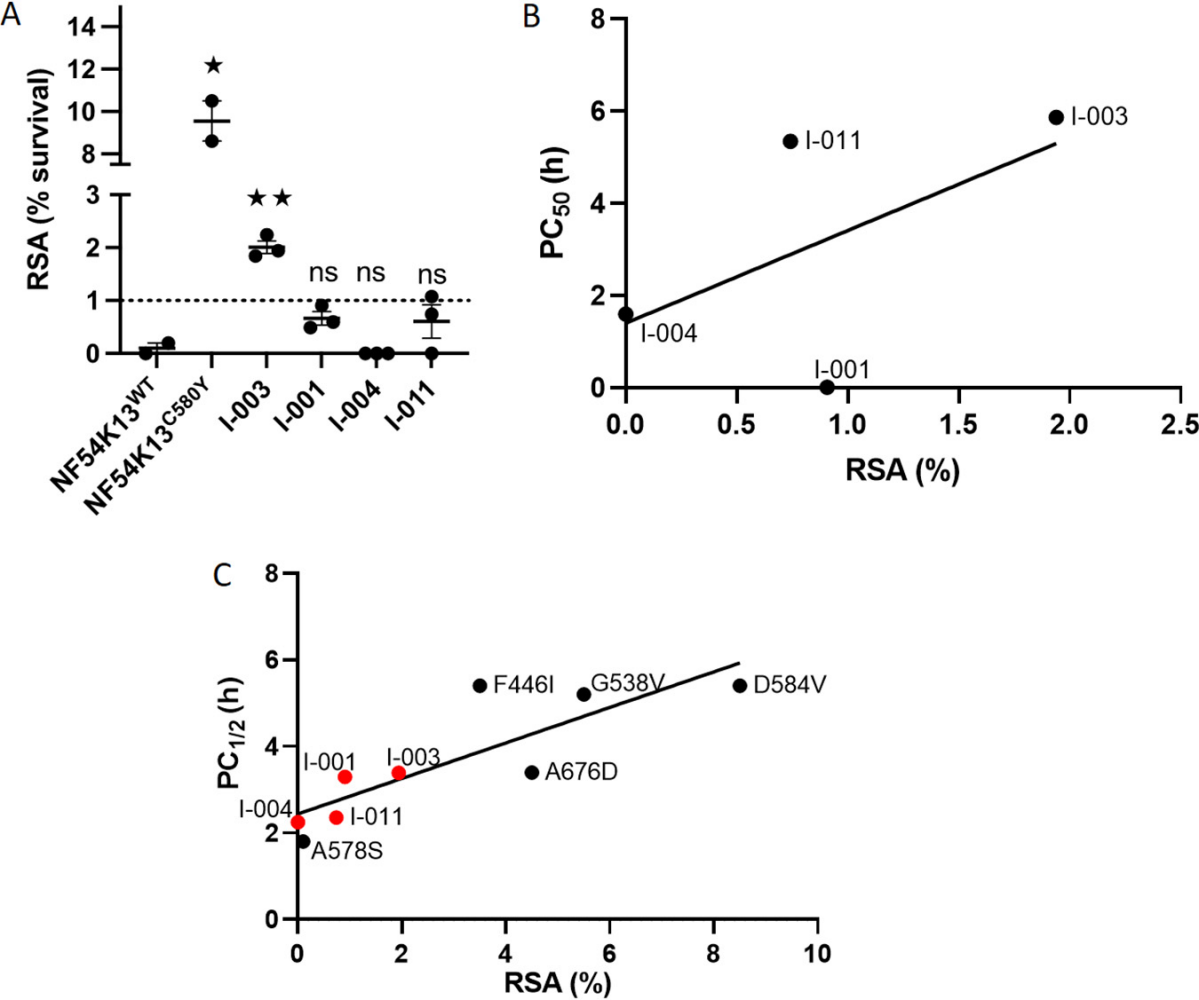
Evaluation of *in vitro* AR by RSA. (A) Survival (%) of four culture-adapted lines in the ring-stage survival assay (RSA_0–3h_). Parasites at 0 to 3 hpi were treated with a 6-h pulse of 700 nM DHA. The drug was washed out, and the final parasitemia was measured by microscopy 66 h later (see Materials and Methods). Mean ± SEM of results of 3 independent biological replicates tested in technical duplicate for Bangladesh strains and 2 biological replicates and technical duplicates for control NF54K13^WT^ and NF54K13^C580Y^ are shown. A dotted line shows the threshold of AR at 1%. NF54K13^WT^ was used as a baseline comparator for all strains in Student's *t* test. *, *P* = 0.01; **, *P* = 0.0016; ns, not significant. (B) Linear regression and correlation analysis of RSA (%) and 50% parasite clearance (PC_50_) (h) of strains I-001, I-003, I-004, and I-011 (*R*^2^ = 0.31). Spearman coefficient, ρ = 0.4, not significant. (C) Linear regression and correlation analysis of RSA (%) and slope half-life (h) of strains I-001, I-003, I-004, and I-011 (red dots) and WHO-validated resistance mutations F446I (Myanmar), A675V (Uganda), G538V (Thai-Myanmar border), and D584V (Cambodia), with slope half-life (PC_1/2_) of <5.5 h (black dots) and RSA assessed with field strains. *R*^2^ = 0.7. Spearman coefficient, ρ = 0.9, *P* < 0.005.

Of the 6 culture-adapted isolates of 2018, isolate O-012 could not be further expanded for RSA studies. Due to insufficient data points, I-002 could not be analyzed by the WWARN PCE tool. Thus, the remaining four isolates, I-001, I-003, I-004, and I-011, were successfully expanded for *in vitro* RSA measurements to the artemisinin derivative, dihydroartemisinin (DHA). As shown in Fig. 5A (and Table S3), the sensitive lab strain NF54 failed to show increased ring-stage survival, while its resistant counterpart, NF54K13^C580Y^, showed significant survival in the presence of DHA, with a mean RSA value of 9.55%. Of the Bangladesh strains, I-004 showed no ring-stage survival. I-001 and I-011 showed RSA values greater than zero but less than 1%, suggesting the absence of AR. However, I-003 displayed RSA values of 2.007% ± 0.119% (mean ± SEM). Since an RSA value of >1% is recognized to signify resistance, our data suggest that I-003 presents a low (compared to NF54K13^C580Y^) but measurable level of AR.

10.1128/mbio.03444-21.4 TABLE S3 Ring-stage survival assay (RSA) data for Bangladesh isolates and control strains. RSA %, percentage parasite survival at 0 to 3 hpi of Bangladeshi isolates treated for 6 h with 700 nM DHA relative to DMSO-treated controls; parasitemia was estimated by microscopy 66 h later. N, number of independent biological replicates. Each biological replicate was performed in technical duplicate. *P* value, unpaired two-tailed *t* tests; ns, nonsignificant. Download Table S3, PDF file, 0.3 MB. Copyright © 2022 Nima et al. 2022 Nima et al. https://creativecommons.org/licenses/by/4.0/ This content is distributed under the terms of the Creative Commons Attribution 4.0 International license.

Since RSAs were designed to mimic the plasma exposure seen during *in vivo* infections (17), we examined the relationship of the RSA to time taken to clear half the initial parasites (data presented in Fig. 4). As shown in Fig. 5B, I-003 demonstrated the greatest time taken for clearance. However, no statistically significant correlation to RSA could be inferred from analyses of all four Bangladesh strains (Fig. 5B). This is likely due to the small numbers; to mitigate this situation, we also examined the correlation between RSA value and half-life of the slope of parasite clearance (PC_1/2_, the major correlate for AR in single-dose, artemisinin monotherapy studies). Since the PC_1/2_ values for Bangladesh strains were below 5.5 h (Tables S4 and S5), we used as comparators published data for PC_1/2_ of <5.5 h of well-established K13 resistance mutations. As shown in Fig. 5C, the data yielded a Spearman correlation coefficient of 0.9 and a *P* value of 0.0014, with an *R*^2^ of 0.71, suggesting that metadata comparisons may flag early stages of emergence of resistance that may otherwise escape standard surveillance.

## DISCUSSION

The goal of Bangladesh’s National Strategic Plan for Malaria Elimination 2017–2021 is to eliminate the disease by 2030 (25). A key component of the plan is to prevent emergence of P. falciparum strains resistant to artemisinins. This needs to be accomplished in addition to interrupting local transmissions and preventing reestablishment of infection. Measurement of *in vivo* clearance of parasites in response to ACT treatment in malaria patients and establishment of culture-adapted Bangladesh strains to determine parasite-intrinsic resistance using RSA are both critically important to guide strategies and interventions to achieve and sustain elimination. It is imperative to understand whether one or both expand over time. Hence, this study fills an important gap in knowledge and provides approaches for identifying the emergence of AR in the CHTs, where >90% of malaria occurs in Bangladesh.

In our study, P. falciparum caused ∼80% to 90% of infections in Bandarban, consistent with prior reports (26). Acute, uncomplicated monoinfections by P. falciparum represented 45% and 39% of patients in 2018 and 2019, respectively (but only about two-thirds of those had parasite densities of >1,000/μL). From 2018 to 2019, malaria had an average of 60% resurgence across Bandarban. Infections rose in the low-API subdistrict of Bandarban Sadar as well as in Alikadam, which has a high API. The increase was well correlated with climatic factors, suggesting that it may be due to increasing local transmission, which is a malaria control priority that must be considered in conjunction with emerging AR (27). These data confirm that although malaria is well controlled in the plains in Bangladesh, it remains endemic with active transmission in the CHTs. We did not collect isolates near the camps of forcibly displaced Myanmar nationals because the camps are located in Cox’s Bazar and not the highlands.

Initial parasite densities of >1,000 parasites/μL and time point samples taken every 6 h for the first 24 h after the administration of the first ACT dose were critical to analyzing the data using the WWARN PCE tool. At the outset of the study, after the first draw at 6 h, subsequent collection time points were spaced 12 h apart in the first 24 to 30 h. But only 60% of these collections could be evaluated by the PCE tool. Upon a shift to collection every 6 h, 100% of collections yielded interpretable data. Across 24 patients, the median time to clear half the initial parasites was 5.6 h, but as many as one-fifth showed a much greater median of 8 h. There was no evidence of drug failure either during the first 72 h in the clinic or the following 30 days at home. There was no correlation between the time to 50% of the initial parasite density (PC_50_) and initial parasitemia or blood cell counts. Although platelet levels were significantly reduced relative to parasitemia (not shown), they showed no correlation with PC_50_. In the case of initial Hb, lymphocytes, and neutrophils, although the correlation coefficient was low (<0.6), the *P* values were <0.05, suggesting that there may be weak correlation that needs further evaluation in subsequent studies. In prior work, correlates of immunity have been inversely correlated with delayed clearance (16). In future studies, measuring the blood cell count every 6 h during the first 24 h of parasite clearance is expected to capture data most pertinent to the window for delayed clearance of parasites *in vivo*. At the time of recruitment, no patient was on antimalarials. But we had no information on whether a patient had had a prior course of artemisinins, which would also be useful to obtain in future work.

Previous studies have reported on analyses of K13 polymorphisms in Bangladesh (21, 28). A single *in vivo* parasite clearance study using single-dose artesunate monotherapy was conducted in the CHTs and published in 2012 (29). An additional single *in vivo* parasite clearance study (15) also using single-dose artesunate monotherapy was undertaken in Ramu in Cox’s Bazar (where malaria is low) but not in the Hill Tracts. Cox’s Bazar reported a median (range) slope of parasite clearance half-life (PC_1/2_) of 2.5 (0.7 to 5.4) between 6 and 24 h after administration. Unlike many studies that report K13 polymorphism with either RSA or parasite clearance data (30–32), we established both, because it was important to undertake correlative analyses between RSA values and slope half-lives of <5.5 h and to obtain insight that the RSA value is a better indicator of low levels of AR than PC_1/2_. Notably, RSA values of >1.0% are recognized by WHO to signify resistance and low RSA values ranging from 1 to 4 are consequential. K13 mutations show different RSA values in different genetic backgrounds; thus, where available, values obtained by gene editing of the Dd2 strain are utilized by WHO. The P574L mutation, 1 of 10 with WHO-validated resistance, yields an RSA value of 1.5% (2). F446I, the second most prevalent mutation in Myanmar engineered into lab strain P. falciparum Dd2, shows an RSA value of ∼2.0% (33). A675V, a recently recognized K13 mutation of AR in Africa, yields an RSA value of 3.5% (34).

Since the Alikadam subdistrict clinic facility lacks facilities for overnight stay, *in vivo* parasite clearance measurements were not undertaken there. But, Alikadam was selected as a second site because it has a subdistrict clinic (Alikadam Upazila Health Complex, with 30 beds to monitor day patients), an API much higher than that of Bandarban (21.4 compared to 0.8 in 2018 and 32.1 compared to 1.6 in 2019), and a closer location to the border with Myanmar, where K13 resistance mutations have been reported and work there enabled rapid collection of infected blood samples for analyses of K13 polymorphisms. Building the requisite clinical capacity will render Alikadam a superior subdistrict site for *in vivo* delayed clearance studies to broaden the comprehensive assessment of AR across remote rural, forested regions with low population density, which present frontiers of malaria in Bangladesh, and other countries where malaria is endemic.

## MATERIALS AND METHODS

### Site selection to support study design.

This is an ongoing prospective study for assessing *in vivo* parasite clearance to determine the presence of clinical AR and its quantitative correlation with parasitemia, comprehensive blood count parameters, and AR molecular markers as well as laboratory adaptation of patient isolates to conduct further *in vitro* and genetic studies of P. falciparum parasite populations in Bandarban. In 2018, we established the clinical capacity to measure *in vivo* AR in Bandarban Sadar Hospital. The site was chosen because it is the only hospital in the CHTs where it is possible to undertake a protocol for *in vivo* delayed P. falciparum clearance, as recommended by the WWARN (23, 35). For this protocol, WWARN recommends obtaining multiple patient blood draws over a 72-h period. The procedure therefore necessitates clinical facilities for inpatient stay for consecutive nights. This is only available at Bandarban Sadar Hospital, because it is the sole district-level hospital in Bandarban District that has all the required facilities. Studies were conducted from 2018 to 2019. They could not be continued in 2020 due to the COVID-19 pandemic.

To reach our target number of isolates for screening K13 polymorphisms (see “Sample size calculation”), in 2019 we added collection of parasite isolates at Alikadam Upazila Health Complex because of its higher annual parasitological index (API; number of malaria cases per thousand individuals per year) (Alikadam, 21.8 in 2018 and 32.1 in 2019; Bandarban, 0.8 in 2018 and 1.6 in 2019).

### Sample size calculation.

Since the absence of substantial information on K13 polymorphisms and no data for *in vivo* clearance in Bangladesh limited the local information on which to base power calculations, we used findings from neighboring Myanmar, which reports a 20% prevalence rate of the F446I mutation (36), causing extended development of the ring stage and some loss of fitness. We assumed the same prevalence in Bandarban for all point mutations in the propeller domain of the K13 gene, with 10% detected difference (precision), 80% power, and 95% level of significance. On this basis, we calculated a sample size of 126, which was also expected to be within clinical capacity and existing information on patient enrollment.

### Patient screening.

Patients were screened when they presented with a positive malaria diagnosis by microscopy and/or RDT. The inclusion criteria were P. falciparum monoinfection, axillary temperature of ≥37.5°C or history of fever during the past 24 h, ability and willingness to comply with the study protocol for the duration of the study, informed consent from the patient or a parent or guardian in the case of children, and minimum age of children of ≥1 year. The exclusion criteria included severe malaria according to the WHO definition (11), i.e., organ dysfunction, multiple convulsions, and impaired consciousness. The exclusion criteria additionally included severe malnutrition, anemia (Hb <8 g/dL), self-administered antimalarial or antibiotics before enrollment, or pregnancy.

### Parasite clearance study procedure.

The initial parasite density and species identification were determined using 200 white blood cells (WBC; thick smear) or 2,000 red blood cells (RBC; thin smear with duplicate slides stained with Giemsa) (Merck, Germany), prior to administration of the first dose of artemether-lumefantrine (A-L) combination therapy (ACT) (Komefan 140; Mylan Laboratories, Ltd., India). The smear results were counted in duplicate by two independent microscopists and then averaged. Parasite density/mL was calculated by dividing the number of asexual parasites by the number of WBC counted (200) and multiplying by an assumed WBC count of 8,000/μL in thick film (or thin film, if the thick film had >250 parasites/50 WBC). Smears were considered negative when no asexual parasites were found after counting 200 WBC.

The admitted indoor patient department (IPD) patients were treated by the hospital staff as described in the protocol of the National Malaria Elimination Program. Briefly, one to four tablets (depending on the patient’s body weight) of A-L (20 mg artemether and 120 mg lumefantrine) was administered at each of the following six time points: 0, 8, 24, 36, 48, 60, and 72 h. Blood draws were planned at 6, 12, 24, 36, and 48 h after the first drug administration (35). Due to logistical difficulties, at the outset, the 12- and 24-h time points were replaced, respectively, by 18- and 36-h time points. However, some patients showed a rapid reduction of parasites in the 12-h interval between 6 h and 18 h, which limited fitting of the data to the curve. For this reason, the first four blood draws were undertaken every 6 h after administration of the first ACT (namely, at 6, 12, 18, and 24 h), followed by a time point collection every 12 h at 36, 48, 60, and 72 h.

Patients were confirmed to be free of parasites and fever for at least 1 day and up to 72 h before being released from the clinic. Smears were considered negative when no asexual parasites were found after counting 200 WBC or 2,000 RBC. Patients were subsequently followed up for fever, pulse, and blood pressure and malarial infection (by RDT and slides) up to 30 days at home weekly, on days 9, 16, 23, and 30. No abnormalities were found. The study had no influence on the treatment decisions made by the hospital physicians.

### Analyses of parasite clearance data.

Parasite clearance times (PCTs) were determined for each patient using the WWARN parasite clearance estimator (PCE) tool (23). The parasite clearance half-life was determined by plotting serial parasite counts on the parasite clearance estimator tool by WWARN (23, 35). The following parameters were defined: clearance rate constant, the fraction by which the parasite count falls per unit time; slope half-life (PC_1/2_), estimated time in hours it takes for the parasitemia to decrease by half (50%), independent of initial parasite density; PC_50,_ PC_90,_ PC_95_, and PC_99_, estimated time in hours for parasitemia to be reduced by 50%, 90%, 95%, and 99% of its initial value (initial parasite density), respectively.

All definitions above follow WWARN methodology for calculating parasite clearance (23, 35).

### Blood cell assessment.

A Sysmex XP-300 hematology analyzer (Sysmex Corporation, Kobe, Japan) was used to provide a full blood cell count, including differential white blood cell counts, prior to administration of the ACT.

### Culture adaptation of patient isolates.

Ten milliliters of venous blood was drawn, before administration of ACT, from adults (≥18 years of age) and 7 mL from adolescents (ages 11 to 17 years) using K_2_EDTA vacutainers (7.2 mg; 4.0 mL) (Becton Dickinsonâ, Erembodegem, Belgium). For culture adaptation, washed infected blood was adjusted to 4% hematocrit in 5 mL RPMI 1640 medium containing l-glutamine, 50 mg/L hypoxanthine, 25 mM HEPES, 2 g/L glucose, 20 μg/mL gentamicin, 0.225% NaHCO_3_, and 15% human pooled plasma with 0.5% Albumax, and cultures were placed in a candle jar (gas conditions, 5% CO_2_, 5% to 10% O_2_, and 85% to 90% N_2_) at 37°C. Cultures were fed and monitored daily: at 2% rings, they were frozen for storage. An adaptation was considered unsuccessful if no parasites emerged up to 60 days.

### RSA.

Parasites were studied by ring-stage survival assay (RSA) after culture adaptation. The RSA_0–3h_ was performed as previously described (20). Briefly, parasites were tightly synchronized through two rounds of sorbitol treatment separated by 40 h. Subsequently, Percoll-purified late-stage segmented schizonts were cultured with fresh RBC for 3 h, followed by sorbitol treatment. Cultures with 0- to 3-h rings were adjusted to 2% hematocrit and 1% parasitemia and seeded into a 24-well plate with 1 mL complete medium per well containing either dihydroartemisinin (DHA) at 700 nM or 0.1% dimethyl sulfoxide (DMSO) (vehicle control). After 6 h at 37°C, the cultures were washed and placed in drug-free medium; after 66 h (72 h from seeding), thin blood smears were prepared and survival was measured microscopically by counting the proportion of next-generation viable rings with normal morphology. Parasitemias were determined by estimating parasitemia from 10,000 RBC. Survival rates (% resistant) were determined by multiplying the ratios of viable parasitemias in DHA-exposed parasites to DMSO-treated controls by 100 and are expressed as a percentage.

### DNA extraction.

Genomic DNA was extracted from 200 μl whole blood using a QiaAmp blood minikit (Qiagen, Inc., Germany).

### Species confirmation by genotyping.

Malaria *Plasmodium* species was confirmed by using a nested PCR in accordance with a previously published method with minor modifications (22).

### K13 propeller domain sequence.

The sequence of the propeller domain of the *kelch13* gene was amplified by nested PCR adapted from previously described procedures (13, 22). An 849-bp product was amplified by ExoSAP-IT (Affymetrix). Amplified products were sequenced using the Sanger sequencing method on an ABI 3500 genetic analyzer (Life Technologies, USA). Sequencing primers included K13_N1_F (5′-GCC AAG CTG CCA TTC ATT TG-3′) and K13_N1_R (5′-GCC TTG TTG AAA GAA GCA GA-3′). Both forward and reverse strands were aligned against P. falciparum 3D7 strain (PF3D7_1343700; PlasmoDB release 46) using CLC Sequence Viewer 8 (Qiagen Aarhus A/S). The sequence of the Bangladesh strain I-003 K13 gene (compared to *Pf3D7*) is provided in Data Set S1 in the supplemental material.

### Weather data.

Weather data for 2018 and 2019 were obtained from the Soil Resource Development Institute (SRDI), Bandarban, Bangladesh.

### Ethical approval.

This study was approved by the Ethical Review Committee of icddr,b (protocol no. PR-17078) and the Institutional Review Board of the University of Notre Dame (protocol no. 17-10-4146).

### Maps.

ArcGIS 10.4.1 (ESRI Inc., Redlands, CA) was used to generate the malaria incidence map for 2019 (Fig. 1A).

### Statistical analysis.

Associations between two variables were assessed using Spearman correlation and Mann-Whitney analysis. All statistical analyses were performed using GraphPad Prism 7 (GraphPad Software, La Jolla, CA).

### Data availability.

All data are available in the supplemental material.
