# Supplementary material for: Assessment of Plasmodium falciparum Artemisinin Resistance Independent of kelch13 Polymorphisms and with Escalating Malaria in Bangladesh
Source: mBio. 2022 Jan 25;13(1):e03444-21. doi: 10.1128/mbio.03444-21 (PMC8787467; doi:10.1128/mbio.03444-21)
Supplement: DATA SET S1 [file mbio.03444-21-sd001.pdf]

Supplementary Information (SI-1)

Patient ID	Year	Collection site	Sex	PC50 (h)	Parasite density (p/μl)	RBC (/μl)	Parasitemia (%)	Hb% (g/dL)	WBC-TC x10^3 (/μL)	Platelets x 10^3 (/μL)	Lymphocytes (%)	Neutrophils (%)	MXD (%)	HCT (%)	MCV (fl)	MCH (pg)	MCHC (g/dL)	Reticulocytes (%)	K13 polymorphisms	Slope half life (h)
001	2018	Bandarban	Female	0.015	2360	3780000	0.062	9.4	5.9	74	36.6	58.3	5.1	26	68.8	24.9	36.2	N/A	no	3.3
002	2018	Bandarban	Male	3.56	165000	5940000	2.778	15.6	6.8	142	8.5	74.9	16.6	47.5	80	26.3	32.8	N/A	no	-
003	2018	Bandarban	Female	5.967	22800	3920000	0.582	10.7	7.8	94	8.4	86.7	4.9	30.9	78.8	27.3	34.6	0.9	no	3.39
004	2018	Bandarban	Male	1.6	3700	6210000	0.060	13	6.7	121	28.2	66.5	5.3	38.2	62.3	21.2	34	N/A	no	2.25
005	2018	Bandarban	Male	3.82	15140	5150000	0.294	14.5	2.7	37	16.1	78.9	5	43	83.5	28.7	34.4	1.03	no	2.49
006	2018	Bandarban	Male	N/A	8600	7000000	0.123	17.7	7.5	84	32.2	48.8	19	52.8	75.4	26	34.5	0.84	no	-
007	2018	Bandarban	Male	N/A	17760	5700000	0.312	13.8	5.6	165	32.2	48.8	19	52.8	75.4	26	34.5	0.79	no	-
008	2018	Bandarban	Male	2.71	140000	4870000	2.875	11.1	5.1	146	30.7	47.2	22.1	33.2	68.2	22.8	33.4	1.03	no	1.42
009	2018	Bandarban	Male	N/A	3920	6280000	0.062	13.7	8	86	19.8	67	13.2	45.3	72.1	21.8	30.2	0.9	no	-
010	2018	Bandarban	Female	2.01	13820	6070000	0.228	11.9	4.3	213	30.1	38.4	31.5	36.5	60.1	19.6	32.6	1.3	no	1.42
011	2018	Bandarban	Male	5.35	207500	5240000	3.960	15.2	5.2	41	15.7	80.8	3.5	44	84	29	34.5	0.88	no	2.36
012	2018	Bandarban	Male	N/A	31840	4700000	0.677	11.6	4.8	46	47.2	37.5	15.3	34.5	73.4	24.7	33.6	0.68	no	-
013	2018	Bandarban	Male	7.98	25200	4880000	0.516	11.2	5.4	40	23.3	63.9	12.8	33.5	68.6	23	33.4	0.52	no	1.56
014	2018	Bandarban	Female	N/A	256250	4940000	5.187	13.3	2.9	29	9.7	83.6	6.7	39.4	79.8	26.9	33.8	0.72	no	-
015	2019	Bandarban	Female	N/A	4480	4910000	0.091	10.8	5.3	126	22.9	65	12.1	32.3	65.8	21.4	32.5	0.8	no	-
016	2019	Bandarban	Male	10.25	20660	7100000	0.291	13.9	12.1	114	9.7	85.3	5	42.8	60.3	13.6	32.5	1	no	1.77
017	2019	Bandarban	Female	4.51	187500	6160000	3.044	13.6	3.7	54	20.9	70.8	8.3	40.8	66.2	22.1	33.3	0.6	no	1.83
018	2019	Bandarban	Male	3.42	195000	6210000	3.140	12.5	7.2	161	18	0	0	37.4	61.4	20.5	33.4	0.4	no	1.86
019	2019	Bandarban	Male	2.02	15920	5610000	0.284	11.2	6.5	67	14.3	75.8	9.9	35.2	62.7	20.1	32.1	1.6	no	2.65
020	2019	Bandarban	Male	7.66	228750	6930000	3.301	16.4	9.5	48	9.1	85.4	5.5	47.5	68.5	22.8	33.3	0.8	no	2.14
021	2019	Bandarban	Female	2.83	37160	4310000	0.862	9.6	4.1	62	38.3	56.2	5.5	31.4	72.9	24.8	34.1	0.8	no	1.22
022	2019	Bandarban	Female	N/A	6980	5550000	0.126	12.2	4.2	102	23.3	55	21.7	38.3	69	22	31.9	0.5	no	-
023	2019	Bandarban	Male	N/A	2500	6810000	0.037	14.8	9.4	181	14.7	79.3	6	46.2	67.8	21.7	32	0.6	no	-
024	2019	Bandarban	Male	N/A	14840	6450000	0.230	15.1	5.8	193	12.5	79.9	7.6	46	71.1	23.3	32.8	1	no	-
025	2019	Bandarban	Male	3.39	1220	6110000	0.020	11.9	6.4	430	18.6	74	7.4	36.5	59.7	19.5	32.6	0.8	no	2.43
026	2019	Bandarban	Male	3.25	20020	3720000	0.538	9.6	5.2	21	45.4	48.4	6.2	27.7	74.5	25.8	34.7	1.8	no	1.62
027	2019	Bandarban	Male	3.23	2940	6030000	0.049	10.3	7.3	86	57.5	31.4	11.1	33.2	55.1	17.1	31	0.6	no	2.89
028	2019	Bandarban	Male	N/A	46100	5700000	0.809	10.7	7.4	120	42	55	3	32.9	54.8	17.5	31.9	0.5	no	-
029	2019	Bandarban	Female	7.68	40940	3200000	1.279	10.1	4.5	110	47	50	3	29.8	66.1	22	33.2	0.7	no	2.39
030	2019	Bandarban	Male	8.19	116875	5250000	2.226	12.5	8.4	81	13	77.9	9.1	29.8	65.9	21.9	33.2	1.40	no	2.04
031	2019	Bandarban	Female	3.41	7900	4550000	0.174	11.8	7.1	144	27	70	3	40.3	82.8	27.1	32.8	0.90	no	1.52
032	2019	Bandarban	Female	N/A	8480	4580000	0.185	12.5	4.8	121	34.4	53.7	11.9	36.5	79.7	27.3	34.2	1.10	no	-
033	2019	Bandarban	Male	8.48	9840	6060000	0.162	11	7.8	141	32	65	3	34	57	18	32	1.40	no	2.42
034	2019	Bandarban	Female	N/A	7740	5450000	0.142	10.1	14.3	476	15.7	77.7	6.6	32.6	59.8	18.5	31	1.20	no	-
035	2019	Bandarban	Female	1.25	13320	3880000	0.343	9.3	4.9	63	31.5	62.4	6.1	27.5	70.9	24	33.8	1.10	no	3.25
036	2019	Bandarban	Male	9.42	9640	5400000	0.179	10.8	8.9	104	6.4	85.4	8.2	32.8	60.7	19.1	31.4	0.60	no	2.69
037	2019	Bandarban	Male	N/A	16940	6300000	0.269	13.1	6.4	51	38	62	0	38.6	61.3	20.8	33.9	0.80	no	-
038	2019	Bandarban	Male	N/A	3480	6160000	0.056	15.8	5.8	74	47	52	1	49.3	80	25.6	32	1.30	no	-
039	2019	Bandarban	Male	13.14	47480	5230000	0.908	13.7	4.1	22	37	58	5	40	76.5	26.2	34.3	0.90	no	2.62
040	2019	Bandarban	Male	3.14	10960	4980000	0.220	10.5	2.2	10.5	19.7	67.2	13.1	31.1	63.1	21.3	33.8	1.20	no	2.17
041	2019	Bandarban	Female	4.82	17780	5310000	0.335	14.1	5.2	53	9	77.7	13.3	41.5	78.2	26.6	34	0.90	no	2.47
001	2019	Alikadam	Female	N/A	26520	-	-	9.4	-	-	-	-	-	-	-	-	-	-	no	-
002	2019	Alikadam	Female	N/A	6320	-	-	13.3	-	-	-	-	-	-	-	-	-	-	no	-
003	2019	Alikadam	Male	N/A	130000	-	-	11.6	-	-	-	-	-	-	-	-	-	-	no	-
004	2019	Alikadam	Male	N/A	21680	-	-	14.5	-	-	-	-	-	-	-	-	-	-	no	-
005	2019	Alikadam	Male	N/A	280	-	-	11.5	-	-	-	-	-	-	-	-	-	-	no	-
006	2019	Alikadam	Male	N/A	47500	-	-	13.1	-	-	-	-	-	-	-	-	-	-	no	-
007	2019	Alikadam	Male	N/A	34240	-	-	9.6	-	-	-	-	-	-	-	-	-	-	no	-
008	2019	Alikadam	Female	N/A	4560	-	-	8.5	-	-	-	-	-	-	-	-	-	-	no	-
009	2019	Alikadam	Female	N/A	39120	-	-	10.8	-	-	-	-	-	-	-	-	-	-	no	-
010	2019	Alikadam	Female	N/A	34760	-	-	9.8	-	-	-	-	-	-	-	-	-	-	no	-
011	2019	Alikadam	Male	N/A	29440	-	-	11.4	-	-	-	-	-	-	-	-	-	-	no	-
012	2019	Alikadam	Female	N/A	10320	-	-	12.5	-	-	-	-	-	-	-	-	-	-	no	-
013	2019	Alikadam	Male	N/A	23280	-	-	12.4	-	-	-	-	-	-	-	-	-	-	no	-
014	2019	Alikadam	Male	N/A	4960	-	-	14.8	-	-	-	-	-	-	-	-	-	-	no	-
015	2019	Alikadam	Male	N/A	15120	-	-	13.6	-	-	-	-	-	-	-	-	-	-	no	-
016	2019	Alikadam	Female	N/A	24920	-	-	10.9	-	-	-	-	-	-	-	-	-	-	no	-
017	2019	Alikadam	Female	N/A	335000	-	-	10.7	-	-	-	-	-	-	-	-	-	-	no	-
018	2019	Alikadam	Male	N/A	3480	5200000	0.067	16	5.8	240	31	58	11	-	-	-	-	-	no	-
019	2019	Alikadam	Male	N/A	600	4900000	0.012	14.4	4.8	190	36	55	9	-	-	-	-	-	no	-
020	2019	Alikadam	Male	N/A	5000	6100000	0.082	19.5	8	235	32	63	5	-	-	-	-	-	no	-
021	2019	Alikadam	Male	N/A	26560	5800000	0.458	17.7	6.2	185	27	64	9	-	-	-	-	-	no	-
022	2019	Alikadam	Female	N/A	4360	4000000	0.109	9.3	10.5	230	20	75	5	-	-	-	-	-	no	-
023	2019	Alikadam	Female	N/A	18560	3800000	0.488	8	7.8	180	47	49	4	-	-	-	-	-	no	-
024	2019	Alikadam	Male	N/A	110000	5400000	2.037	15.7	5.6	150	16	78	6	-	-	-	-	-	yes	-
025	2019	Alikadam	Female	N/A	280	4100000	0.007	10.6	7.5	260	43	52	5	-	-	-	-	-	no	-
026	2019	Alikadam	Male	N/A	105000	4500000	2.333	11.6	6.9	190	21	72	7	-	-	-	-	-	no	-
027	2019	Alikadam	Male	N/A	23240	4600000	0.505	12.5	9.1	210	25	68	7	-	-	-	-	-	no	-
028	2019	Alikadam	Male	N/A	50000	5500000	0.909	15.1	4.3	135	47	45	8	-	-	-	-	-	no	-
029	2019	Alikadam	Male	N/A	15280	5900000	0.259	16.4	9.5	290	14	83	3	-	-	-	-	-	no	-
030	2020	Alikadam	Male	N/A	2040	3900000	0.052	8.3	7.3	175	33	58	9	-	-	-	-	-	no	-
031	2020	Alikadam	Male	N/A	372500	4100000	9.085	10.2	9.1	155	30	63	7	-	-	-	-	-	no	-
032	2020	Alikadam	Male	N/A	7760	3500000	0.222	8.5	7.9	170	20	70	10	-	-	-	-	-	no	-
033	2020	Alikadam	Male	N/A	122500	5400000	2.269	15.5	4.9	130	16	78	6	-	-	-	-	-	no	-
031 (Re)	2020	Alikadam	Male	N/A	167500	4900000	3.418	13.4	8.7	183	25	69	6	-	-	-	-	-	no	-
034	2020	Alikadam	Male	N/A	17520	4200000	0.417	12.2	7.2	339	18	75	7	-	-	-	-	-	no	-
035	2020	Alikadam	Male	N/A	310000	4400000	7.045	12.2	10.5	390	21	65	14	-	-	-	-	1.2	no	-
036	2020	Alikadam	Male	N/A	3920	4900000	0.080	12.3	10.2	170	29	61	10	-	-	-	-	0.9	no	-
037	2020	Alikadam	Female	N/A	2680	5300000	0.051	12.5	5.8	145	23	69	8	-	-	-	-	0.6	no	-
038	2020	Alikadam	Male	N/A	1315000	4800000	27.396	11.8	9.8	190	13	82	5	-	-	-	-	0.8	no	-
039	2020	Alikadam	Female	N/A	2280	3600000	0.063	9.8	4.2	285	46	50	4	-	-	-	-	0.4	no	-
040	2020	Alikadam	Male	N/A	2120	5100000	0.042	14.6	6.1	160	20	75	5	-	-	-	-	1.4	no	-
041	2020	Alikadam	Male	N/A	322500	4300000	7.500	11.4	4.7	155	44	52	4	-	-	-	-	0.6	no	-
042	2020	Alikadam	Male	N/A	20120	3400000	0.592	10.8	6.1	170	23	73	4	-	-	-	-	1	no	-
043	2020	Alikadam	Male	N/A	45000	4100000	1.098	13.8	4.2	295	38	52	10	-	-	-	-	0.7	no	-
044	2020	Alikadam	Male	N/A	17960	4900000	0.367	14.4	10.2	185	18	79	3	-	-	-	-	0.5	no	-
045	2020	Alikadam	Female	N/A	7360	3800000	0.194	9.7	4.9	150	39	55	6	-	-	-	-	0.7	no	-
046	2020	Alikadam	Male	N/A	82500	3400000	2.426	9.3	8.2	340	21	71	8	-	-	-	-	1.4	no	-
047	2020	Alikadam	Male	N/A	105000	5500000	1.909	15	6.5	220	19	75	4	-	-	-	-	0.8	no	-
048	2020	Alikadam	Male	N/A	337500	4000000	8.438	11.2	6.1	155	22	75	3	-	-	-	-	1.6	no	-
049	2020	Alikadam	Female	N/A	23760	4600000	0.517	11.8	8.1	190	9	88	3	-	-	-	-	0.4	no	-
050	2020	Alikadam	Female	N/A	4200	2600000	0.162	8.1	6.3	370	28	68	4	-	-	-	-	1.8	no	-
051	2020	Alikadam	Female	N/A	8200	4300000	0.191	12.2	5.5	175	23	72	5	-	-	-	-	0.5	no	-
052	2020	Alikadam	Male	N/A	2040	4000000	0.051	15	4.8	160	38	56	6	-	-	-	-	0.9	no	-
053	2020	Alikadam	Male	N/A	190000	4800000	3.958	15	4.1	135	13	84	3	-	-	-	-	0.6	no	-
054	2020	Alikadam	Male	N/A	117500	3300000	3.561	9.1	6.7	155	15	80	5	-	-	-	-	1.4	no	-
055	2020	Alikadam	Male	N/A	280	5500000	0.005	13.8	5.9	160	11	85	4	-	-	-	-	0.9	no	-
056	2020	Alikadam	Male	N/A	45000	3700000	1.216	11.6	5.1	165	34	60	6	-	-	-	-	0.5	no	-
057	2020	Alikadam	Male	N/A	90000	4300000	2.093	13.5	6.5	150	10	86	4	-	-	-	-	0.8	no	-
058	2020	Alikadam	Female	N/A	2440	3100000	0.079	9.9	4.1	185	46	48	6	-	-	-	-	1.2	no	-
059	2020	Alikadam	Male	N/A	32360	5700000	0.568	15.9	6.2	170	32	60	8	-	-	-	-	1	no	-
060	2020	Alikadam	Male	N/A	4880	3500000	0.139	9.4	8.2	210	55	39	6	-	-	-	-	2.2	no	-
061	2020	Alikadam	Male	N/A	21920	4000000	0.548	12.4	3.5	110	82	16	2	-	-	-	-	0.8	no	-
062	2020	Alikadam	Male	N/A	277500	4400000	6.307	11	5.9	155	25	71	4	-	-	-	-	1.1	no	-
063	2020	Alikadam	Male	N/A	4760	5400000	0.088	14.8	6.5	175	30	67	3	-	-	-	-	1.8	no	-
064	2020	Alikadam	Male	N/A	147500	4100000	3.598	12.3	9.1	160	27	69	4	-	-	-	-	2.4	no	-
065	2020	Alikadam	Male	N/A	117500	5200000	2.260	14.4	8.5	185	23	71	6	-	-	-	-	2	no	-
066	2020	Alikadam	Male	N/A	2600	3900000	0.067	11.2	6.2	205	19	76	5	-	-	-	-	1	no	-
067	2020	Alikadam	Female	N/A	2480	2700000	0.092	8.5	6.7	140	39	57	4	-	-	-	-	0.6	no	-
068	2020	Alikadam	Male	N/A	1440	3500000	0.041	12.1	5.5	320	29	65	6	-	-	-	-	0.8	no	-
069	2020	Alikadam	Male	N/A	25000	4300000	0.581	14.8	3.7	180	42	55	3	-	-	-	-	2.8	no	-
070	2020	Alikadam	Female	N/A	172500	3300000	5.227	11.1	6.1	160	35	61	4	-	-	-	-	0.5	no	-
071	2020	Alikadam	Female	N/A	152500	3500000	4.357	10.5	7.5	220	21	77	2	-	-	-	-	0.7	no	-
072	2020	Alikadam	Male	N/A	3000	3200000	0.094	10.9	11.2	190	30	67	3	-	-	-	-	0.5	no	-
073	2020	Alikadam	Male	N/A	1120	3600000	0.031	11.7	5.7	175	38	59	3	-	-	-	-	1.2	no	-
074	2020	Alikadam	Male	N/A	142500	4800000	2.969	15.7	3.1	90	45	52	3	-	-	-	-	0.6	no	-
075	2020	Alikadam	Female	N/A	107500	4000000	2.688	13.3	8.2	270	24	69	7	-	-	-	-	0.8	no	-
076	2020	Alikadam	Male	N/A	5120	4900000	0.104	11	4	170	42	50	8	-	-	-	-	1	no	-
077	2020	Alikadam	Male	N/A	72500	4600000	1.576	14.2	6.3	160	14	84	2	-	-	-	-	1.6	no	-
078	2020	Alikadam	Female	N/A	70000	4500000	1.556	12.5	4.9	195	11	86	3	-	-	-	-	1	no	-
079	2020	Alikadam	Male	N/A	205000	2900000	7.069	8.4	7.7	170	50	45	5	-	-	-	-	0.5	no	-
080	2020	Alikadam	Male	N/A	112500	3600000	3.125	10.6	6.5	130	35	59	6	-	-	-	-	0.6	no	-
081	2020	Alikadam	Female	N/A	15520	4200000	0.370	11.8	7.5	190	10	86	4	-	-	-	-	2.6	no	-
082	2020	Alikadam	Male	N/A	127500	4900000	2.602	15.8	5.1	255	51	43	6	-	-	-	-	1.8	no	-
083	2020	Alikadam	Female	N/A	307500	3100000	9.919	10.6	6.8	150	19	78	3	-	-	-	-	0.8	no	-
084	2020	Alikadam	Male	N/A	285000	3900000	7.308	13.2	5.5	180	14	84	2	-	-	-	-	1	no	-
085	2020	Alikadam	Male	N/A	97500	2900000	3.362	10.4	5.8	120	25	70	5	-	-	-	-	0.4	no	-

Supporting Information (SI-2)

I-003 full length Kelch13 sequence aligned to Pf3D7

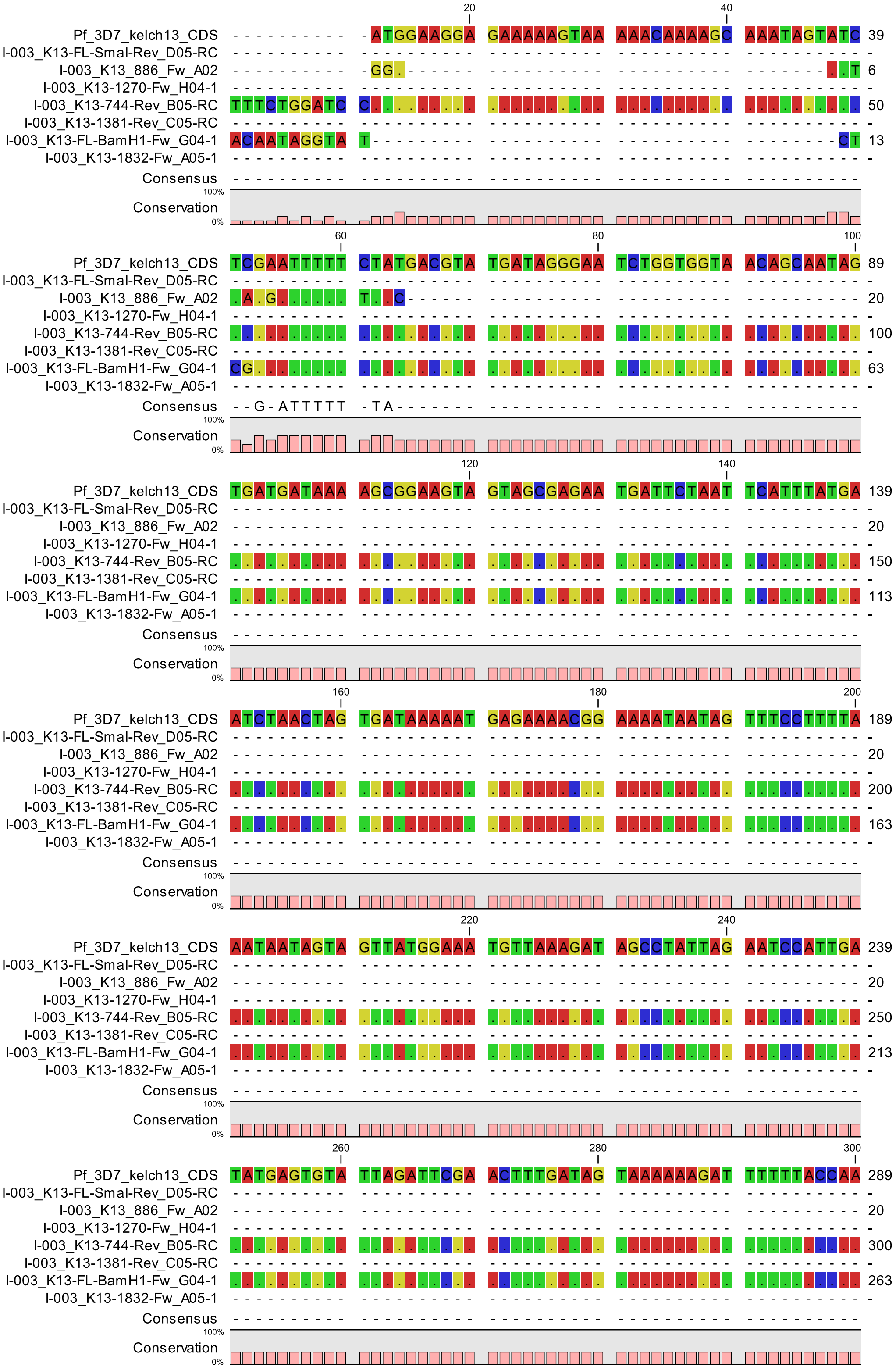

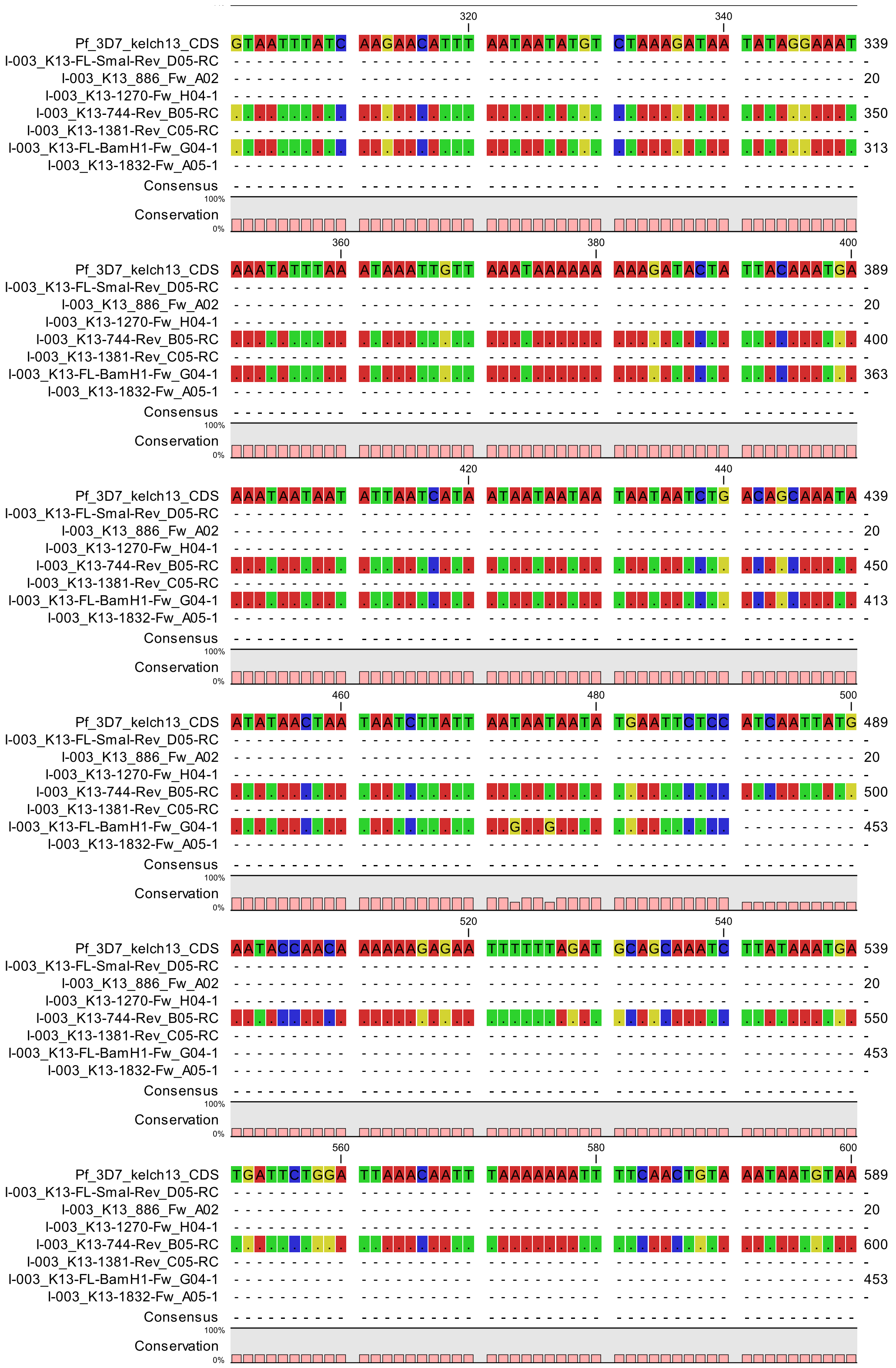
Pf_3D7_kelch13_CDS
I-003_K13-FL-SmaI-Rev_D05-RC
I-003_K13_886_Fw_A02
I-003_K13-1270-Fw_H04-1
I-003_K13-744-Rev_B05-RC
I-003_K13-1381-Rev_C05-RC
I-003_K13-FL-BamH1-Fw_G04-1
I-003_K13-1832-Fw_A05-1
Consensus
Conservation
100%
0%
GTAATTTATC AAGAACATTT AATAATATGT CTAAAGATAA TATAGGAAAT 339
AAATATTTAA ATAAATTGTT AAATAAAAAA AAAGATACTA TTACAAATGA 389
AAATAATAAT ATTAATCATA ATAATAATAA TAATAATCTG ACAGCAAATA 439
ATATAACTAA TAATCTTATT AATAATAATA TGAATTCTCC ATCAATTATG 489
AATACCAACA AAAAAGAGAA TTTTTTAGAT GCAGCAAATC TTATAAATGA 539
TGATTCTGGA TTAAACAATT TAAAAAAATT TTCAACTGTA AATAATGTAA 589

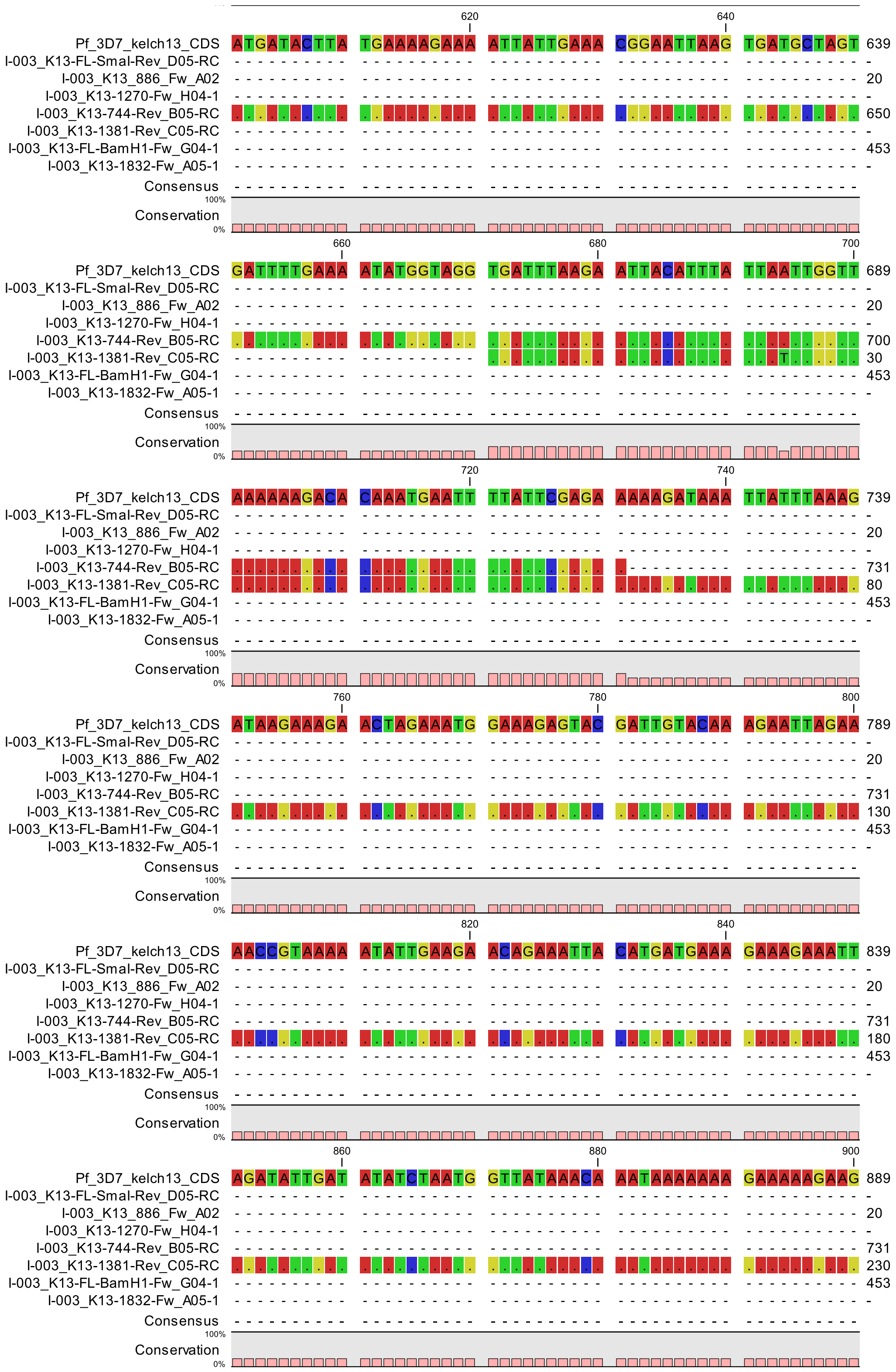

620
640
Pf_3D7_kelch13_CDS ATGATACTTA TGAAAAGAAA ATTATTGAAA CGGAATTAAG TGATGCTAGT 639
I-003_K13-FL-SmaI-Rev_D05-RC -
I-003_K13_886_Fw_A02 20
I-003_K13-1270-Fw_H04-1 -
I-003_K13-744-Rev_B05-RC 650
I-003_K13-1381-Rev_C05-RC -
I-003_K13-FL-BamH1-Fw_G04-1 453
I-003_K13-1832-Fw_A05-1 -
Consensus
Conservation
100%
0%
660
680
700
Pf_3D7_kelch13_CDS GATTTTGAAA ATATGGTAGG TGATTTAAGA ATTACATTTA TTAATTGGTT 689
I-003_K13-FL-SmaI-Rev_D05-RC -
I-003_K13_886_Fw_A02 20
I-003_K13-1270-Fw_H04-1 -
I-003_K13-744-Rev_B05-RC 700
I-003_K13-1381-Rev_C05-RC T 30
I-003_K13-FL-BamH1-Fw_G04-1 453
I-003_K13-1832-Fw_A05-1 -
Consensus
Conservation
100%
0%
720
740
Pf_3D7_kelch13_CDS AAAAAAGACA CAAATGAATT TTATTCGAGA AAAAGATAAA TTATTTAAAG 739
I-003_K13-FL-SmaI-Rev_D05-RC -
I-003_K13_886_Fw_A02 20
I-003_K13-1270-Fw_H04-1 -
I-003_K13-744-Rev_B05-RC 731
I-003_K13-1381-Rev_C05-RC 80
I-003_K13-FL-BamH1-Fw_G04-1 453
I-003_K13-1832-Fw_A05-1 -
Consensus
Conservation
100%
0%
760
780
800
Pf_3D7_kelch13_CDS ATAAGAAAGA ACTAGAAATG GAAAGAGTAC GATTGTACAA AGAATTAGAA 789
I-003_K13-FL-SmaI-Rev_D05-RC -
I-003_K13_886_Fw_A02 20
I-003_K13-1270-Fw_H04-1 -
I-003_K13-744-Rev_B05-RC 731
I-003_K13-1381-Rev_C05-RC 130
I-003_K13-FL-BamH1-Fw_G04-1 453
I-003_K13-1832-Fw_A05-1 -
Consensus
Conservation
100%
0%
820
840
Pf_3D7_kelch13_CDS AACCGTAAAA ATATTGAAGA ACAGAAATTA CATGATGAAA GAAAGAAATT 839
I-003_K13-FL-SmaI-Rev_D05-RC -
I-003_K13_886_Fw_A02 20
I-003_K13-1270-Fw_H04-1 -
I-003_K13-744-Rev_B05-RC 731
I-003_K13-1381-Rev_C05-RC 180
I-003_K13-FL-BamH1-Fw_G04-1 453
I-003_K13-1832-Fw_A05-1 -
Consensus
Conservation
100%
0%
860
880
900
Pf_3D7_kelch13_CDS AGATATTGAT ATATCTAATG GTTATAAACA AATAAAAAAA GAAAAAGAAG 889
I-003_K13-FL-SmaI-Rev_D05-RC -
I-003_K13_886_Fw_A02 20
I-003_K13-1270-Fw_H04-1 -
I-003_K13-744-Rev_B05-RC 731
I-003_K13-1381-Rev_C05-RC 230
I-003_K13-FL-BamH1-Fw_G04-1 453
I-003_K13-1832-Fw_A05-1 -
Consensus
Conservation
100%
0%

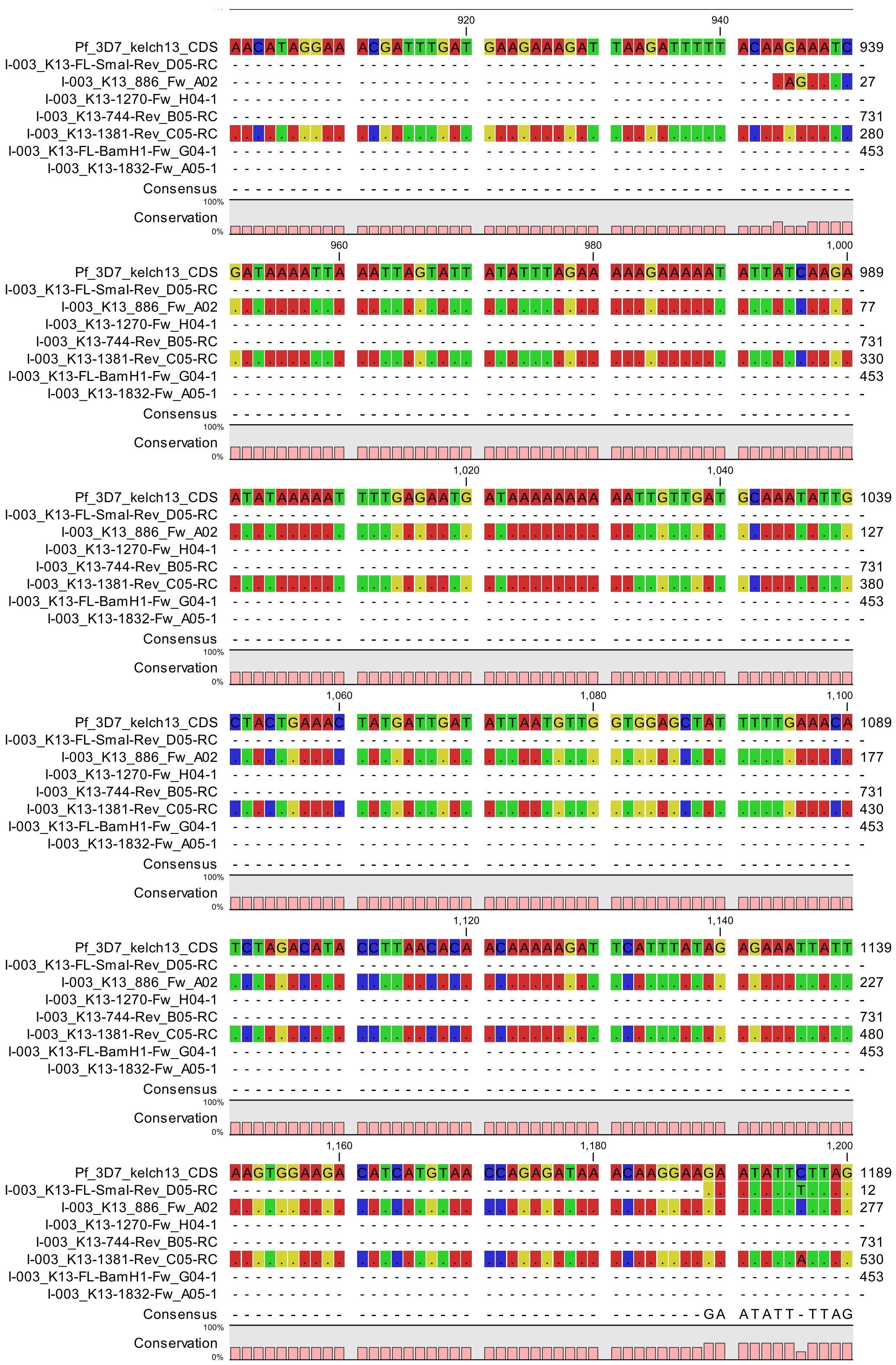
920
940
Pf_3D7_kelch13_CDS AACATAGGAA ACGATTTGAT GAAGAAAGAT TAAGATTTTT ACAAGAAATC 939
I-003_K13-FL-SmaI-Rev_D05-RC -
I-003_K13_886_Fw_A02 27
I-003_K13-1270-Fw_H04-1 -
I-003_K13-744-Rev_B05-RC 731
I-003_K13-1381-Rev_C05-RC 280
I-003_K13-FL-BamH1-Fw_G04-1 453
I-003_K13-1832-Fw_A05-1 -
Consensus
Conservation
960
980
1,000
Pf_3D7_kelch13_CDS GATAAAATTA AATTAGTATT ATATTTAGAA AAAGAAAAAT ATTATCAAGA 989
I-003_K13-FL-SmaI-Rev_D05-RC -
I-003_K13_886_Fw_A02 77
I-003_K13-1270-Fw_H04-1 -
I-003_K13-744-Rev_B05-RC 731
I-003_K13-1381-Rev_C05-RC 330
I-003_K13-FL-BamH1-Fw_G04-1 453
I-003_K13-1832-Fw_A05-1 -
Consensus
Conservation
1,020
1,040
Pf_3D7_kelch13_CDS ATATAAAAAT TTTGAGAATG ATAAAAAAAA AATTGTTGAT GCAAATATTG 1039
I-003_K13-FL-SmaI-Rev_D05-RC -
I-003_K13_886_Fw_A02 127
I-003_K13-1270-Fw_H04-1 -
I-003_K13-744-Rev_B05-RC 731
I-003_K13-1381-Rev_C05-RC 380
I-003_K13-FL-BamH1-Fw_G04-1 453
I-003_K13-1832-Fw_A05-1 -
Consensus
Conservation
1,060
1,080
1,100
Pf_3D7_kelch13_CDS CTACTGAAAC TATGATTGAT ATTAATGTTG GTGGAGCTAT TTTTGAAACA 1089
I-003_K13-FL-SmaI-Rev_D05-RC -
I-003_K13_886_Fw_A02 177
I-003_K13-1270-Fw_H04-1 -
I-003_K13-744-Rev_B05-RC 731
I-003_K13-1381-Rev_C05-RC 430
I-003_K13-FL-BamH1-Fw_G04-1 453
I-003_K13-1832-Fw_A05-1 -
Consensus
Conservation
1,120
1,140
Pf_3D7_kelch13_CDS TCTAGACATA CCTTAACACA ACAAAAAGAT TCATTTATAG AGAAATTATT 1139
I-003_K13-FL-SmaI-Rev_D05-RC -
I-003_K13_886_Fw_A02 227
I-003_K13-1270-Fw_H04-1 -
I-003_K13-744-Rev_B05-RC 731
I-003_K13-1381-Rev_C05-RC 480
I-003_K13-FL-BamH1-Fw_G04-1 453
I-003_K13-1832-Fw_A05-1 -
Consensus
Conservation
1,160
1,180
1,200
Pf_3D7_kelch13_CDS AAGTGGAAGA CATCATGTAA CCAGAGATAA ACAAGGAAGA ATATTCTTAG 1189
I-003_K13-FL-SmaI-Rev_D05-RC 12
I-003_K13_886_Fw_A02 277
I-003_K13-1270-Fw_H04-1 -
I-003_K13-744-Rev_B05-RC 731
I-003_K13-1381-Rev_C05-RC 530
I-003_K13-FL-BamH1-Fw_G04-1 453
I-003_K13-1832-Fw_A05-1 -
Consensus GA ATATT-TTAG
Conservation

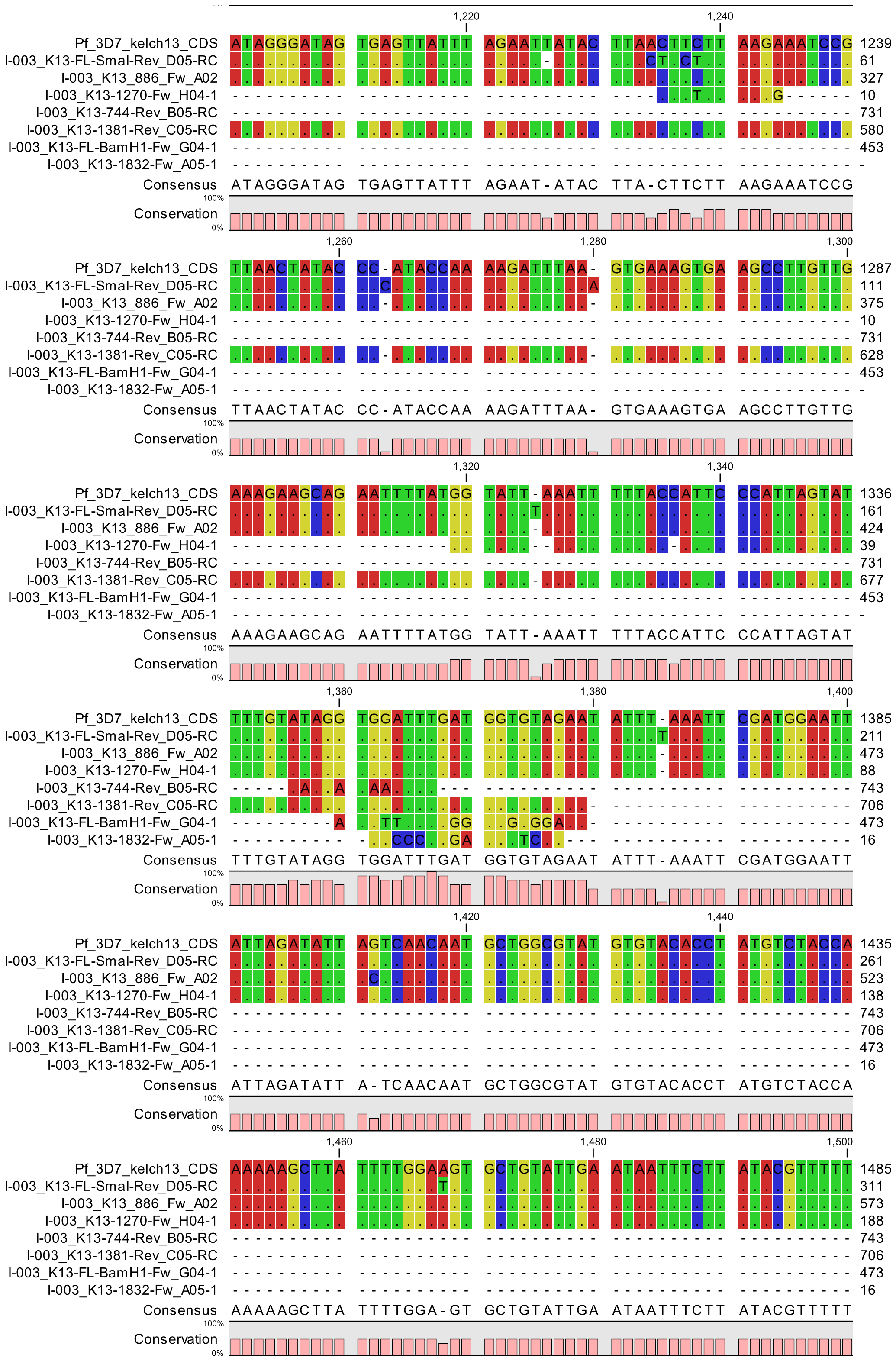
Pf_3D7_kelch13_CDS
I-003_K13-FL-SmaI-Rev_D05-RC
I-003_K13_886_Fw_A02
I-003_K13-1270-Fw_H04-1
I-003_K13-744-Rev_B05-RC
I-003_K13-1381-Rev_C05-RC
I-003_K13-FL-BamH1-Fw_G04-1
I-003_K13-1832-Fw_A05-1
Consensus
Conservation
ATAGGGATAG TGAGTTATTT AGAAT-ATAC TTA-CTTCTT AAGAAATCCG
TTAACTATAC CC-ATACCAA AAGATTTAA- GTGAAAGTGA AGCCTTGTTG
AAAGAAGCAG AATTTTATGG TATT-AAATT TTTACCATTC CCATTAGTAT
TTTGTATAGG TGGATTTGAT GGTGTAGAAT ATTT-AAATT CGATGGAATT
ATTAGATATT A-TCAACAAT GCTGGCGTAT GTGTACACCT ATGTCTACCA
AAAAAGCTTA TTTTGGA-GT GCTGTATTGA ATAATTTCTT ATACGTTTTT

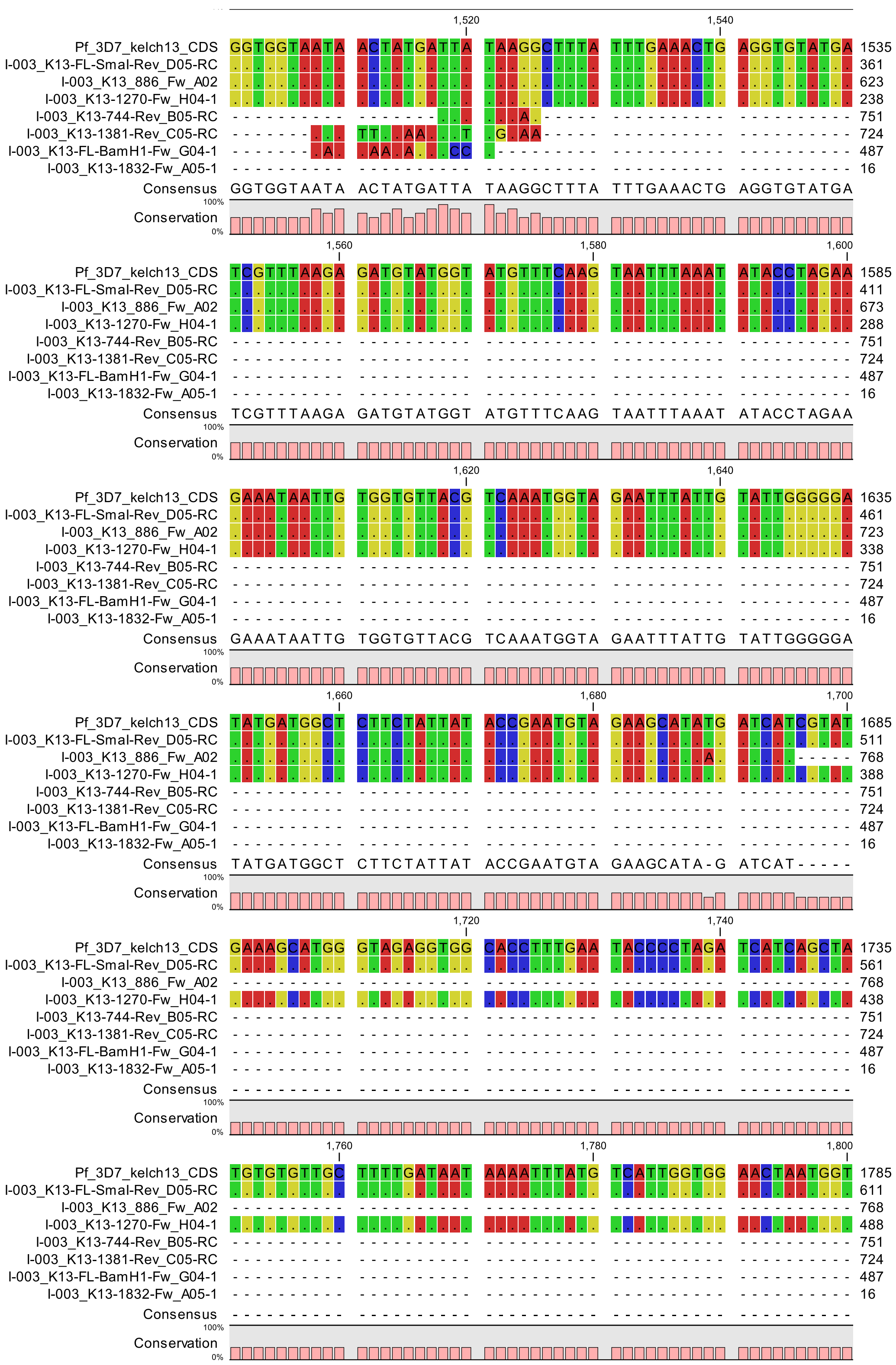
Pf_3D7_kelch13_CDS
I-003_K13-FL-SmaI-Rev_D05-RC
I-003_K13_886_Fw_A02
I-003_K13-1270-Fw_H04-1
I-003_K13-744-Rev_B05-RC
I-003_K13-1381-Rev_C05-RC
I-003_K13-FL-BamH1-Fw_G04-1
I-003_K13-1832-Fw_A05-1
Consensus
Conservation
GGTGGTAATA ACTATGATTA TAAGGCTTTA TTTGAAACTG AGGTGTATGA 1535
TCGTTTAAGA GATGTATGGT ATGTTTCAAG TAATTTAAAT ATACCTAGAA 1585
GAAATAATTG TGGTGTTACG TCAAATGGTA GAATTTATTG TATTGGGGGA 1635
TATGATGGCT CTTCTATTAT ACCGAATGTA GAAGCATATG ATCATCGTAT 1685
TATGATGGCT CTTCTATTAT ACCGAATGTA GAAGCATA-G ATCAT-----
GAAAGCATGG GTAGAGGTGG CACCTTTGAA TACCCCTAGA TCATCAGCTA 1735
TGTGTGTTGC TTTTGATAAT AAAATTTATG TCATTGGTGG AACTAATGGT 1785

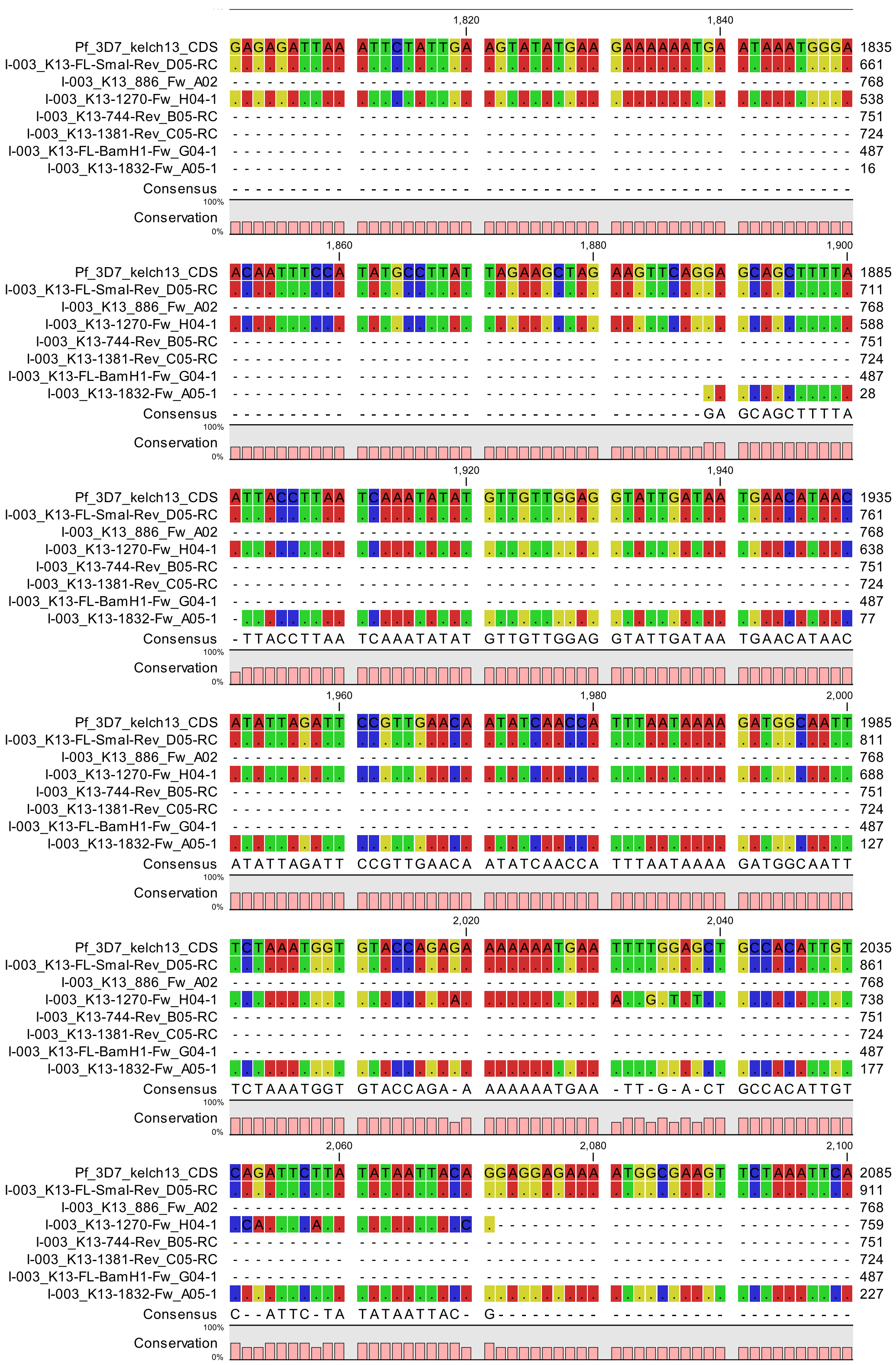
Pf_3D7_kelch13_CDS
I-003_K13-FL-SmaI-Rev_D05-RC
I-003_K13_886_Fw_A02
I-003_K13-1270-Fw_H04-1
I-003_K13-744-Rev_B05-RC
I-003_K13-1381-Rev_C05-RC
I-003_K13-FL-BamH1-Fw_G04-1
I-003_K13-1832-Fw_A05-1
Consensus
Conservation
100%
0%

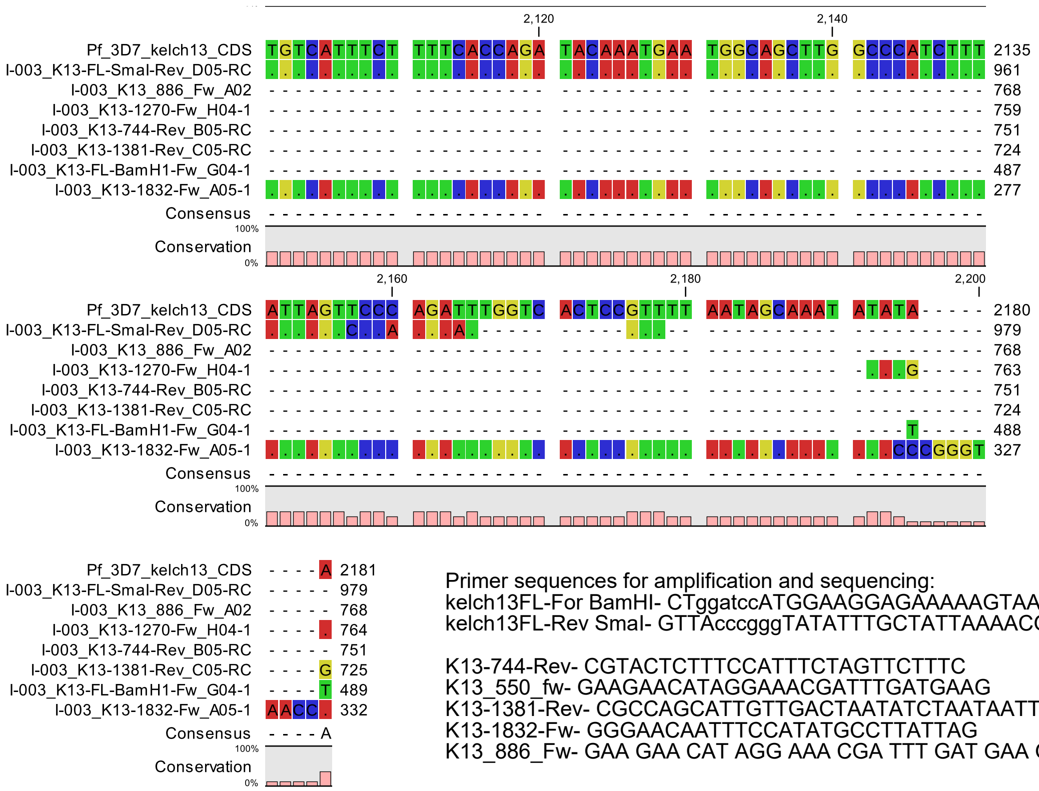

SmaI restriction site: CCCGGG
kelch13FL-Rev SmaI-
GTTAcccgggTATATTTGCTATTAAAACGGAGTGACC

Primer sequences for amplification and sequencing:
kelch13FL-For BamHI- CTggatccATGGAAGGAGAAAAAGTAAAAACAAAAG
kelch13FL-Rev SmaI- GTTAcccgggTATATTTGCTATTAAAACGGAGTGACC

K13-744-Rev- CGTACTCTTTCCATTTCTAGTTCTTTC
K13_550_fw- GAAGAACATAGGAAACGATTTGATGAAG
K13-1381-Rev- CGCCAGCATTGTTGACTAATATCTAATAATTC
K13-1832-Fw- GGGAACAATTTCCATATGCCTTATTAG
K13_886_Fw- GAA GAA CAT AGG AAA CGA TTT GAT GAA G
