# Supplementary material for: Assessment of Plasmodium falciparum Artemisinin Resistance Independent of kelch13 Polymorphisms and with Escalating Malaria in Bangladesh
Source: mBio. 2022 Jan 25;13(1):e03444-21. doi: 10.1128/mbio.03444-21 (PMC8787467; doi:10.1128/mbio.03444-21)
Supplement: TABLE S1 [file mbio.03444-21-st001.pdf]

Table S1.

Patient ID	Year	Age	Gender	Temperature at enrollment (°C)	Initial parasite density (/µl)	Parasitemia %	Hb% (g/dL)
I-001	2018	45	Female	39.4	2360	0.062	9.4
I-002	2018	17	Male	39.4	165000	2.78	15.6
I-003	2018	45	Female	39.4	22800	0.58	10.7
I-004	2018	45	Male	37.6	3700	0.059	13
I-005	2018	missing data	Male	40	15140	0.293	14.5
O-006	2018	26	Male	36	8600	0.122	17.7
O-007	2018	44	Male	35.5	17760	0.311	13.8
I-008	2018	29	Male	38.3	140000	2.874	11.1
O-009	2018	22	Male	38.3	3920	0.062	13.7
I-010	2018	13	Female	37.2	13820	0.227	11.9
I-011	2018	24	Male	36.6	207500	3.959	15.2
O-012	2018	35	Male	38.9	31840	0.677	11.6
I-013	2018	10	Male	37.8	25200	0.516	11.2
O-014	2018	30	Female	39.4	256250	5.187	13.3
O-015	2019	22	Female	38.9	4480	0.091	10.8
I-016	2019	13	Male	40	20660	0.29	13.9
I-017	2019	15	Female	39.4	187500	3.043	13.6
I-018	2019	17	Male	37.7	195000	3.14	12.5
I-019	2019	12	Male	38.3	15920	0.283	11.2
I-020	2019	27	Male	37.8	228750	3.3	16.4
I-021	2019	7	Female	37.4	37160	0.862	9.6
O-022	2019	17	Female	38.9	6980	0.125	12.2
O-023	2019	22	Male	38.9	2500	0.036	14.8
O-024	2019	20	Male	38.3	14840	0.23	15.1
I-025	2019	56	Male	37.2	1220	0.019	11.9
I-026	2019	60	Male	38.3	20020	0.538	9.6
I-027	2019	18	Male	37.8	2940	0.048	10.3
O-028	2019	41	Male	37.7	46100	0.808	10.7
I-029	2019	29	Female	38.3	40940	1.279	10.1
I-030	2019	14	Male	40	116875	2.226	12.5
I-031	2019	11	Female	38.3	7900	0.173	11.8
O-032	2019	40	Female	38.3	8480	0.185	12.5
I-033	2019	16	Male	38.9	9840	0.162	11
O-034	2019	4	Female	40.6	7740	0.142	10.1
I-035	2019	17	Female	38.9	13320	0.343	9.3
I-036	2019	68	Male	40	9640	0.178	10.8
O-037	2019	28	Male	38.3	16940	0.268	13.1
O-038	2019	56	Male	37.8	3480	0.056	15.8

I-039	2019	40	Male	38.9	47480	0.907	13.7
I-040	2019	51	Male	40	10960	0.22	10.5
I-041	2019	30	Female	38.3	17780	0.334	14.1
