# Supplementary figures and images for: Assessment of Plasmodium falciparum Artemisinin Resistance Independent of kelch13 Polymorphisms and with Escalating Malaria in Bangladesh
Source: mBio. 2022 Jan 25;13(1):e03444-21. doi: 10.1128/mbio.03444-21 (PMC8787467; doi:10.1128/mbio.03444-21)

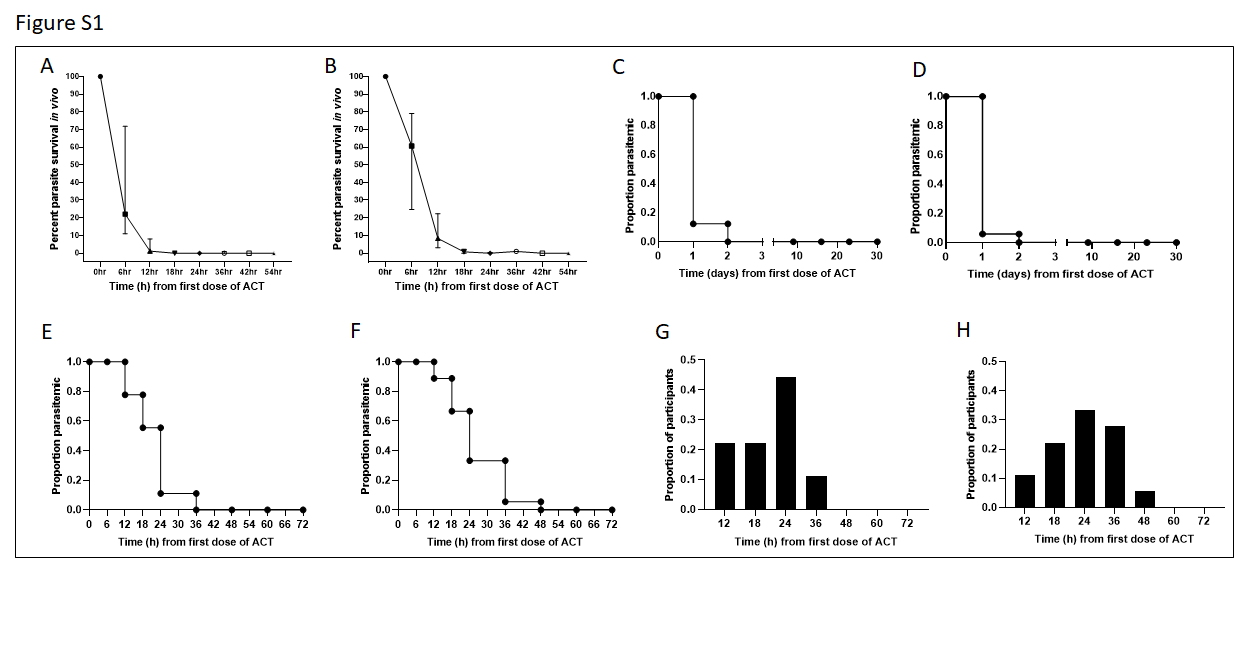

Supplement: FIG S1 [file mbio.03444-21-sf001.tif]

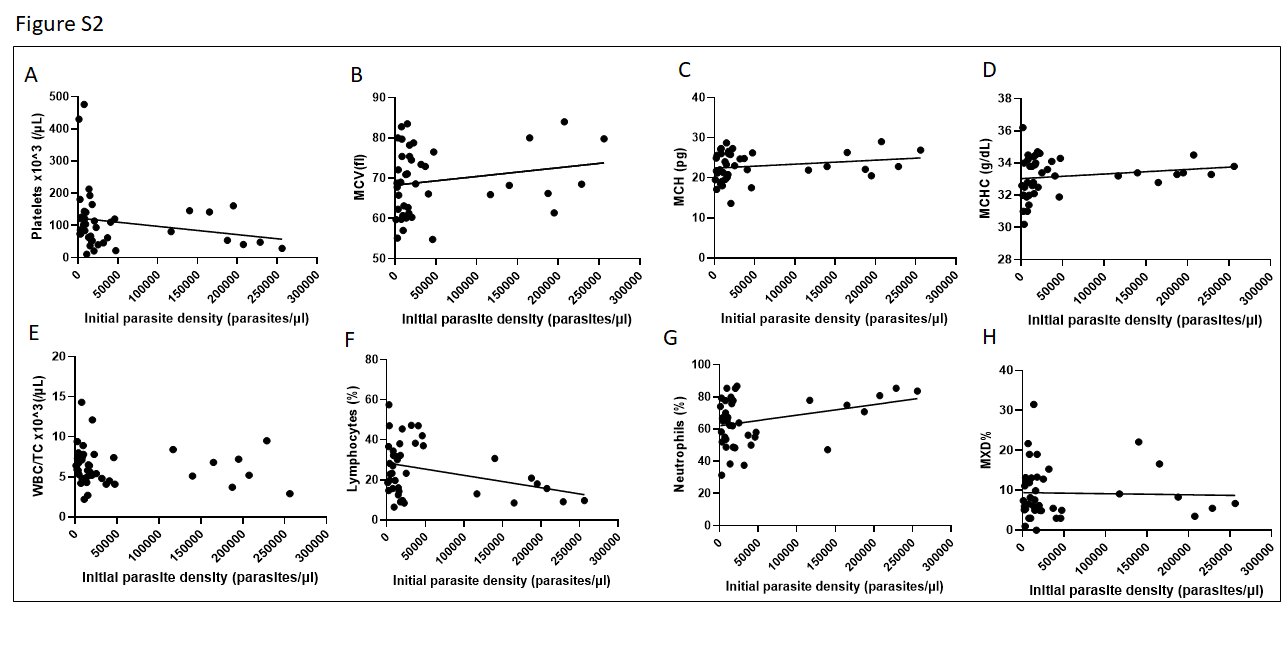

Supplement: FIG S2 [file mbio.03444-21-sf002.tif]

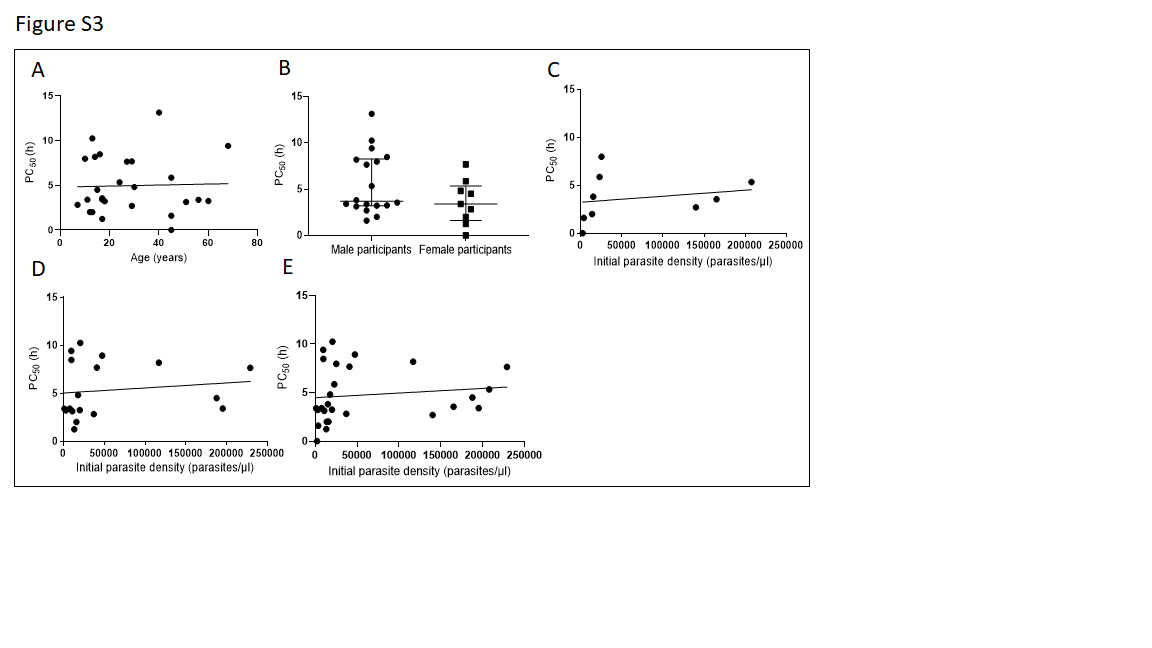

Supplement: FIG S3 [file mbio.03444-21-sf003.tif]

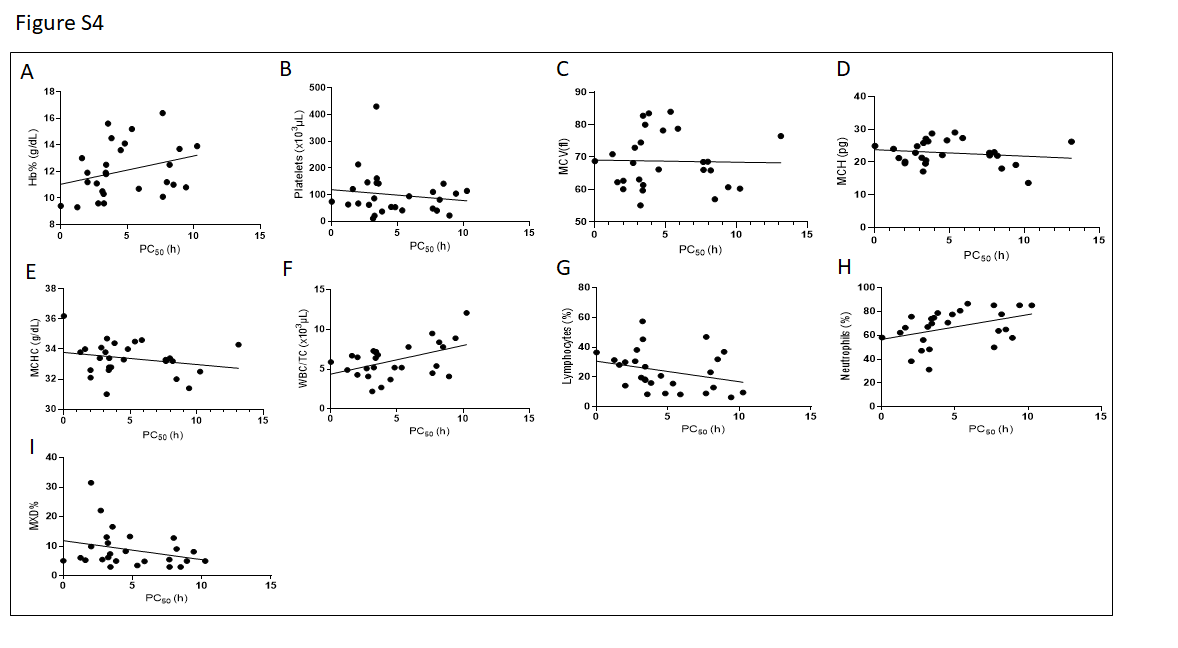

Supplement: FIG S4 [file mbio.03444-21-sf004.tif]

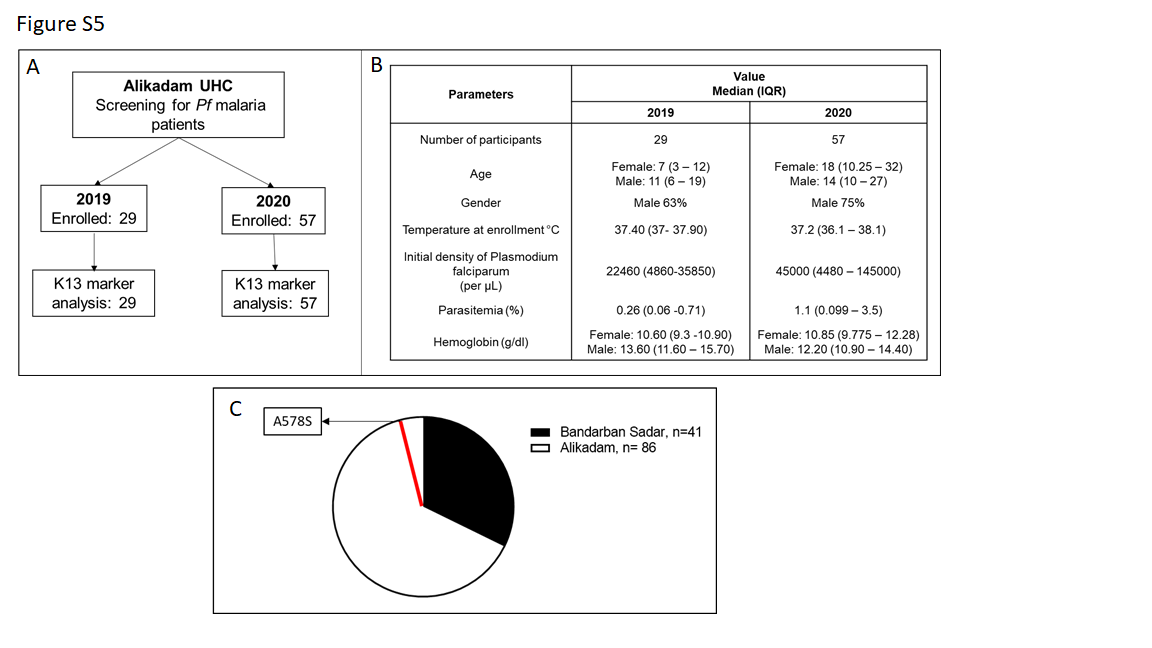

Supplement: FIG S5 [file mbio.03444-21-sf005.tif]

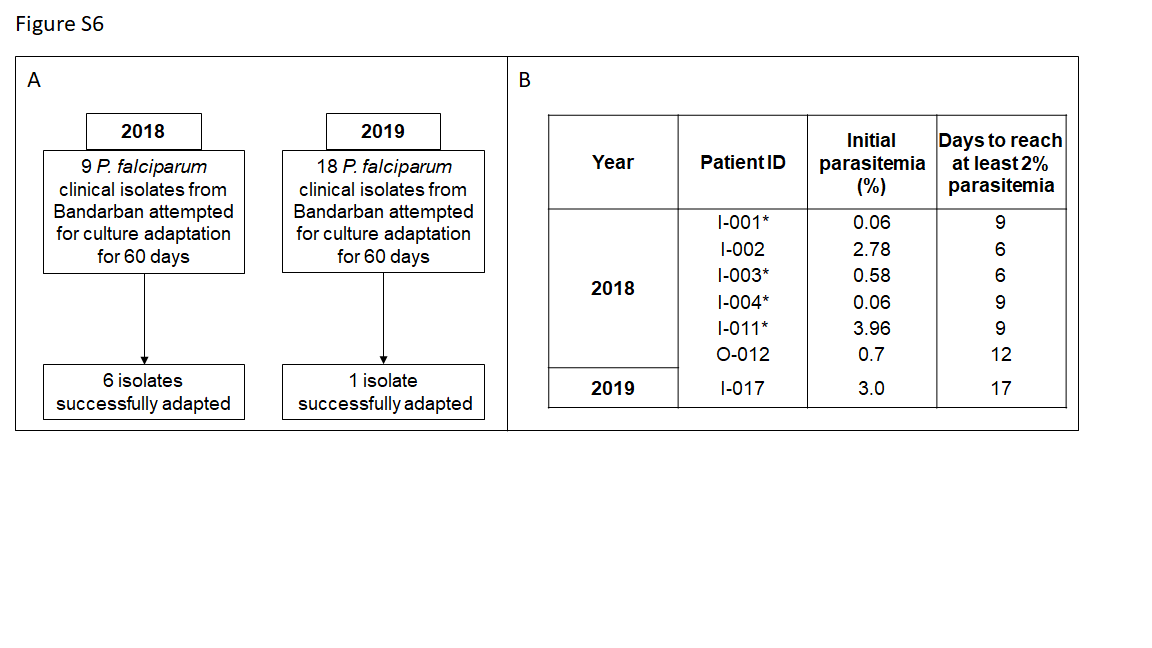

Supplement: FIG S6 [file mbio.03444-21-sf006.tif]
