# Supplementary material for: Assessment of Plasmodium falciparum Artemisinin Resistance Independent of kelch13 Polymorphisms and with Escalating Malaria in Bangladesh
Source: mBio. 2022 Jan 25;13(1):e03444-21. doi: 10.1128/mbio.03444-21 (PMC8787467; doi:10.1128/mbio.03444-21)
Supplement: TABLE S2 [file mbio.03444-21-st002.pdf]

Table S2.

Patient ID	Year	Reticulocytes (%)	Platelets x10^3 (/µL)	MCV (fl)	MCH (pg)	MCHC (g/dL)	WBC/TC x10^3 (/µL)	Lymphocytes (%)	Neutrophils (%)	MXD (%)
I-001	2018	-	74	68.8	24.9	36.2	5.9	36.6	58.3	5.1
I-002	2018	-	142	80	26.3	32.8	6.8	8.5	74.9	16.6
I-003	2018	0.9	94	78.8	27.3	34.6	7.8	8.4	86.7	4.9
I-004	2018	-	121	62.3	21.2	34	6.7	28.2	66.5	5.3
I-005	2018	1.03	37	83.5	28.7	34.4	2.7	16.1	78.9	5
O-006	2018	0.84	84	75.4	26	34.5	7.5	32.2	48.8	19
O-007	2018	0.79	165	75.4	26	34.5	5.6	32.2	48.8	19
I-008	2018	1.03	146	68.2	22.8	33.4	5.1	30.7	47.2	22.1
O-009	2018	0.9	86	72.1	21.8	30.2	8	19.8	67	13.2
I-010	2018	1.3	213	60.1	19.6	32.6	4.3	30.1	38.4	31.5
I-011	2018	0.88	41	84	29	34.5	5.2	15.7	80.8	3.5
O-012	2018	0.68	46	73.4	24.7	33.6	4.8	47.2	37.5	15.3
I-013	2018	0.52	40	68.6	23	33.4	5.4	23.3	63.9	12.8
O-014	2018	0.72	29	79.8	26.9	33.8	2.9	9.7	83.6	6.7
O-015	2019	0.8	126	65.8	21.4	32.5	5.3	22.9	65	12.1
I-016	2019	1	114	60.3	13.6	32.5	12.1	9.7	85.3	5
I-017	2019	0.6	54	66.2	22.1	33.3	3.7	20.9	70.8	8.3
I-018	2019	0.4	161	61.4	20.5	33.4	7.2	18	0	0
I-019	2019	1.6	67	62.7	20.1	32.1	6.5	14.3	75.8	9.9
I-020	2019	0.8	48	68.5	22.8	33.3	9.5	9.1	85.4	5.5
I-021	2019	0.8	62	72.9	24.8	34.1	4.1	38.3	56.2	5.5
O-022	2019	0.5	102	69	22	31.9	4.2	23.3	55	21.7
O-023	2019	0.6	181	67.8	21.7	32	9.4	14.7	79.3	6
O-024	2019	1	193	71.1	23.3	32.8	5.8	12.5	79.9	7.6
I-025	2019	0.8	430	59.7	19.5	32.6	6.4	18.6	74	7.4
I-026	2019	1.8	21	74.5	25.8	34.7	5.2	45.4	48.4	6.2
I-027	2019	0.6	86	55.1	17.1	31	7.3	57.5	31.4	11.1
O-028	2019	0.5	120	54.8	17.5	31.9	7.4	42	55	3
I-029	2019	0.7	110	66.1	22	33.2	4.5	47	50	3
I-030	2019	1.40	81	65.9	21.9	33.2	8.4	13	77.9	9.1
I-031	2019	0.90	144	82.8	27.1	32.8	7.1	27	70	3
O-032	2019	1.10	121	79.7	27.3	34.2	4.8	34.4	53.7	11.9
I-033	2019	1.40	141	57	18	32	7.8	32	65	3
O-034	2019	1.20	476	59.8	18.5	31	14.3	15.7	77.7	6.6
I-035	2019	1.10	63	70.9	24	33.8	4.9	31.5	62.4	6.1
I-036	2019	0.60	104	60.7	19.1	31.4	8.9	6.4	85.4	8.2
O-037	2019	0.80	51	61.3	20.8	33.9	6.4	38	62	0
O-038	2019	1.30	74	80	25.6	32	5.8	47	52	1
I-039	2019	0.90	22	76.5	26.2	34.3	4.1	37	58	5

I-040	2019	1.20	10.5	63.1	21.3	33.8	2.2	19.7	67.2	13.1
I-041	2019	0.90	53	78.2	26.6	34	5.2	9	77.7	13.3
