# Supplementary material for: Assessment of Plasmodium falciparum Artemisinin Resistance Independent of kelch13 Polymorphisms and with Escalating Malaria in Bangladesh
Source: mBio. 2022 Jan 25;13(1):e03444-21. doi: 10.1128/mbio.03444-21 (PMC8787467; doi:10.1128/mbio.03444-21)
Supplement: TABLE S3 [file mbio.03444-21-st003.pdf]

Table S3.

**Parasite**	**K13**	**RSA%[a]**			
		Mean	SEM	N[b]	P value[c]
NF54K13WT	WT	0.1	0.1	2	-
NF54K13C580Y	C580Y	9.55	0.95	2	0.01
I-003	WT	2.007	0.1195	3	0.001
I-001	WT	0.6636	0.126	3	ns
I-004	WT	0	0	3	ns
I-011	WT	0.6058	0.3178	3	ns
